# Antiviral and immunomodulatory effect of zapnometinib in animal models and hospitalized COVID-19 patients

**DOI:** 10.3389/fimmu.2025.1631721

**Published:** 2025-09-22

**Authors:** Yvonne Füll, Lara M. Schüssele, Hazem Hamza, Helen Hoffmann, Martin Bauer, Stephan Stenglein, Oliver Pötz, Andreas Steinhilber, Viktoria Anselm, Mark W. Delany, Judith M. A. Van den Brand, Geert Van Amerongen, Leon De Waal, Stephan Pleschka, Stephan Ludwig, Oliver Planz

**Affiliations:** ^1^ Institute of Immunology, Eberhard Karls University, Tuebingen, Germany; ^2^ Atriva Therapeutics GmbH, Tuebingen, Germany; ^3^ Virology Laboratory, Environmental Research Division, National Research Centre, Cairo, Egypt; ^4^ SIGNATOPE GmbH, Reutlingen, Germany; ^5^ Division of Pathology, Faculty of Veterinary Medicine Utrecht, University Utrecht, Utrecht, Netherlands; ^6^ Viroclinics-DDL, Cerba Research Company, Schaijk, Netherlands; ^7^ Institute of Medical Virology, Justus Liebig University, Giessen, Germany; ^8^ Institute of Virology (IVM), Centre for Molecular Biology of Inflammation, University of Muenster, Muenster, Nordrhein-Westfalen, Germany

**Keywords:** zapnometinib, MEK inhibitor, host targeting agent, COVID-19, drug development, immunomodulation, broad-spectrum antiviral

## Abstract

**Introduction:**

In severe COVID-19, direct-acting antiviral drugs were not effective in hyperinflammatory stages and steroid treatment may weaken host immunity. The MEK inhibitor zapnometinib, as a host-targeting drug, has demonstrated promising efficacy against severe acute viral infections. Proof-of-concept for the innovative approach was presented in a clinical Phase 2 trial with hospitalized COVID-19 patients.

**Methods:**

The antiviral and immunomodulatory potential of zapnometinib was investigated in samples obtained from COVID-19 patients enrolled in a Phase 2 clinical trial (RESPIRE), as well as in a SARS-CoV-2 Syrian hamster model, an acute lung injury mouse model, and in cell culture. The antiviral activity of zapnometinib was assessed using viral load reduction assays and RT-qPCR. Cytokines and chemokines were analyzed via ELISA and RT-qPCR. Alterations in T and B cells from COVID-19 patients were analyzed using flow cytometry. Biomarker analysis in hamster serum was conducted to monitor potential toxic effects.

**Results:**

Zapnometinib reduced SARS-CoV-2 viral load in hospitalized COVID-19 patients, in the hamster model and in various highly pathogenic coronaviruses *in vitro*. Pro-inflammatory cytokines and chemokines decreased in COVID-19 patients, in a lung injury mouse model, and *in vitro* in primary human blood cells treated with zapnometinib. In the hamster model, zapnometinib alleviated SARS-CoV-2-mediated lung pathology. In patients with COVID-19, zapnometinib increased T and plasma B cells.

**Conclusion:**

Unlike direct-acting antivirals, zapnometinib’s dual effect highlights its therapeutic potential in the treatment of severe acute viral infections, with favorable antiviral and immunomodulatory properties.

## 1 Introduction

In late 2019, severe acute respiratory syndrome coronavirus 2 (SARS-CoV-2), which causes coronavirus disease 2019 (COVID-19), emerged in humans. The World Health Organization (WHO) declared COVID-19 a pandemic due to the rapid person-to-person transmission of SARS-CoV-2 and the significant morbidity and mortality it caused worldwide (1–3). The severity of COVID-19 varies widely from asymptomatic infection to severe disease, with the latter being associated with lower respiratory tract symptoms and pathological hyperinflammation (4–6) which may lead to multiorgan dysfunction, including acute respiratory distress syndrome (ARDS), neurological symptoms, cardiovascular events, and coagulopathies (7–9). While the viral load is high at onset and in the early stages of COVID-19, the hyperinflammatory response plays a more important role in determining disease severity in later stages (10). Hyperinflammation is evidenced by significantly elevated levels of certain proinflammatory cytokines and chemokines observed in the majority of COVID-19 patients, particularly in those with severe disease requiring intensive care (11).

Currently available treatments can be categorized into therapeutic antibodies, repurposed direct-acting antivirals (DAA) like nirmatrelvir, remdesivir, or molnupiravir, and immunomodulating drugs, such as dexamethasone or anti-IL-6 antibodies (12–16). They all have their limitations. Therapeutic antibodies have become less effective due to the emergence of new SARS-CoV-2 variants, underscoring the issue of viral escape leading to resistance. Direct-acting antivirals must be administered very early in the onset of infection and have not proven to be highly effective in treating severely ill patients. Various immunomodulatory approaches, some with promising results, were employed for treating lung inflammation in COVID‐19 patients. Examples of these include the intake of probiotics, prebiotics, vitamins, or zinc. However, the appropriate dosage and timing of intake still need to be evaluated, and any potential benefits should clearly outweigh the risks (17–19). Approved immunomodulating drugs such as dexamethasone have demonstrated significant efficacy in advanced stages of infection. Nevertheless, administering them too early could potentially impede the initiation of an antiviral immune response. Thus, a drug that is effective throughout the full course of the disease is still missing. Targeting cellular factors presents a promising avenue for addressing this therapeutic gap. The cellular Raf/MEK/ERK signaling pathway is mainly involved in cell proliferation and regulation of cytokine and chemokine expression (20). Furthermore, the inhibition of this signaling pathway induces a shift in the adaptive immune response towards an effector immune reaction (21–23). Severe cases of COVID-19 are characterized by aberrant adaptive immunity, specifically evidenced by lymphopenia and especially impaired CD8^+^ T cell functionality (24, 25). Consequently, targeting this signaling pathway may ameliorate hyperactive innate and adaptive immune responses.

MEK (mitogen-activated protein kinase kinase) inhibitors prevent the phosphorylation of ERK (extracellular signal-regulated kinase) in cells where the signaling pathway is activated, such as in cancer cells or cells attacked by certain viruses. In most of our somatic cells, the signaling pathway is not highly activated. Several MEK inhibitors are already in development or approved for the treatment of various cancers (26). We previously demonstrated that targeting the cellular Raf/MEK/ERK signaling pathway leads to a reduction in viral load and protects mice from lethal infection with another health-relevant respiratory virus, the influenza A virus (IAV) (27–29). Additionally, apart from IAV, various RNA-viruses, like Respiratory Syncytial virus (RSV), Zika virus (ZIKV), Dengue virus (DENV), Yellow fever virus (YFV), Borna disease virus (BDV) or Mouse hepatitis virus (MHV), require activation of the Raf/MEK/ERK signaling pathway to ensure their propagation. Inhibition of this pathway disrupts various stages of their viral life cycle (27, 30–33). There are also certain viruses that are not sensitive to MEK inhibition (34, 35). This prompted us to analyze whether inhibition of the pathway would also result in inhibition of SARS-CoV-2 replication. Indeed, we were able to demonstrate that zapnometinib (ATR-002, PD 0184264), an oral MEK inhibitor, not only exhibits antiviral activity against the influenza virus in both *in vitro* and *in vivo* settings (28, 29), but also against SARS-CoV-2 *in vitro* in cell lines as well as in primary air-liquid-interphase epithelial cell cultures, with a safe and selective treatment window (36). Furthermore, the *in vitro* synergistic effect of zapnometinib was demonstrated in combination with baloxavir-marboxil against IAV (29), and also with various direct-acting SARS-CoV-2 drugs (37). In the life cycle of IAV, the transport of viral ribonucleoprotein complexes relies on active MEK (27). Despite SARS-CoV-2 lacking a nuclear phase in its replication cycle, MEK activation assumes a crucial function in the initial phases of infection (36). It is noteworthy from the aforementioned study that the immunomodulatory impact of zapnometinib operates autonomously from its antiviral properties. It could be shown that MEK inhibition leads to reduced expression of pro-inflammatory cytokines and chemokines without interfering with the interferon-induced antiviral response (38, 39). In the subsequent stages of drug development, it was possible to demonstrate that zapnometinib showed no toxicity and was well tolerated in humans in two phase 1 studies in healthy volunteers (NCT04385420; EudraCT 2021-005225-25). The correlation between pharmacokinetics, pharmacodynamics and antiviral efficacy could be demonstrated in various animal models and in humans, confirming both safety and efficacy and highlighting the drug’s significant therapeutic potential (40). Moreover, an absorption, distribution, metabolism, and excretion (ADME) study in rats showed a favorable distribution and metabolism of the drug (41). In the phase 2 clinical trial of hospitalized COVID-19 patients (RESPIRE; NCT04776044), zapnometinib treatment was found to be safe and well-tolerated. Consistent trends toward better outcomes were observed with zapnometinib, especially among patients with severe disease (42).

Here, our aim was to evaluate the therapeutic potential of zapnometinib against various coronaviruses, demonstrating its dual effect in a phase 2 clinical trial with hospitalized COVID-19 patients as well as through different pre-clinical approaches. Zapnometinib treatment leads to a reduction in viral load and favorable immunomodulatory effects on top of standard of care. These results are supported by preclinical data from a Syrian hamster SARS-CoV-2 infection model, wherein zapnometinib significantly reduces viral load and lung pathology without causing toxic effects. Furthermore, our findings demonstrate that zapnometinib can significantly alleviate hyperinflammation induced by cytokines and chemokines associated with severe COVID-19 in an acute lung injury (ALI) mouse model and in primary human blood cells.

These findings support the potential of zapnometinib as a promising broad-spectrum antiviral therapeutic with a dual effect, particularly in severe respiratory viral infections leading to hyperinflammation such as COVID-19 or influenza.

## 2 Material and methods

### 2.1 Ethics

The hamster experiments were carried out in the Central Animal Facilities of Viroclinics-DDL, Cerba Research Company, Schaijk, the Netherlands, in accordance with the standards of the Dutch law for animal experimentation (2010/63/EU). Ethical approval for the present study was obtained from the institutional Animal Welfare Body under the number AVD277002015283-WP24.

The mouse experiments for the ALI mouse model were conducted following review and approval by the Regional Council Tuebingen (35/9185.81-2/IM 1/17) and in compliance with the institutional Principles of Animal Welfare and Animal Research.

Human sputum and nasopharyngeal swabs were taken from samples collected for the RESPIRE trial (NCT04776044) in 18 hospitals from 5 countries that was approved by respective regulatory authorities and by the Ethics Committees concerned (for details, please see the clinical trial protocol provided in (42).

### 2.2 Animals

Seven- to nine-week-old male Syrian hamsters (Janvier, France) with a body weight ranging from 102 to 134 g at the time of drug administration were utilized for the pharmacokinetic study. The same animals, aged 11 to 13 weeks and a body weight ranging from 113 to 148 g, were included for the efficacy study. The animals were housed in elongated type 2 IVC (individually ventilated cages) group cages with two animals per cage under BSL-II conditions during acclimatization and in elongated type 2 group cages under BSL-III conditions (isolators) during challenge, with sawdust as bedding. Daily checks were conducted for overt signs of disease, standard food was provided, and drinking water was available ad libitum. All biotechnical procedures were performed under 3% isoflurane anesthesia.

Six- to eight-week-old female C57BL/6J mice weighing 20–27 g from the Mouse Facility of the University of Tuebingen (Interfaculty Institute for Cell Biology) were utilized to establish an inflammatory acute lung injury (ALI) model. The animals were maintained in elongated type 2 IVC cages housing four mice each under BSL-II conditions and provided with standard food and drinking water ad libitum.

### 2.3 Virus and cells

SARS-CoV-2 (BetaCoV/Munich/BavPat1/2020, containing the D614G substitution in the S1 fragment, kindly provided by Dr. C. Drosten, Berlin, Germany) was passaged once in Vero-TMPRSS2 cells and three times in Vero E6 cells and titered in a TCID_50_ assay as described in the section “Determination of replication-competent virus (TCID_50_ assay)”. SARS-CoV-2 “Omicron” (B.1.1.592) (kindly provided by Prof. Dr. M. Schindler, Institute of Medical Virology and Epidemiology, University Hospital Tübingen) was passaged on Calu-3 cells and titrated by plaque assay, as previously described in Koch-Heier et al., 2021 (43). Briefly, Vero E6 cells were infected with a 6-fold serial dilution of the virus stock, overlayed with Avicel medium and incubated for 72h at 37°C, 5% CO_2_. After fixation and staining of the cell layer with crystal violet solution, the virus titer was determined based on the number of plaques counted per well and the respective dilution factor. Vero E6 (Cat# CRL-1586), Vero cells (Cat# CCL-81), MRC-5 (Cat# CCL-171) and Calu-3 cells (Cat# HTB-55) used for *in vitro* assays were obtained from ATCC (Manassas, Virginia, USA). The virus strains used for *in vitro* studies at a MOI of 0.001 were the following: SARS-CoV-1: SARS-CoV-HKU-39849; SARS-CoV-2 BavPat1: Human 2019-nCoV Isolate, EVAg Ref-SKU:026V-03883; SARS-CoV-2 “Omicron” B.1.1.592 (described in (44):); HCoV-229E (Cat# VR-740; ATCC). HCoV-OC43 (Cat# VR-1558; ATCC) was used at MOI 0.1.

Human peripheral blood mononuclear cells (PBMCs) were isolated from healthy volunteers registered with the Biobank of the Department of Immunology at the University of Tuebingen, after informed consent documented in writing. Ethical approval was obtained upon review by the Ethics Board of the Medical Faculty of the Eberhard Karls University Tuebingen and the University Hospital Tuebingen (887/2020BO2). Briefly, 50 ml whole blood (roughly 5x10^7^ PBMCs) of two donors were taken in 50 ml syringes (BD Biosciences, Franklin Lakes, New Jersey, USA) supplied with heparin-natrium (100.000 I.E./ml; Braun, Melsungen, Germany). The whole blood was mixed with PBS (Gibco, Carlsbad, California, USA) in a 1:1 ratio in 50 ml tubes (Greiner Bio-One, Frickenhausen, Germany), and PBMCs were subsequently isolated from the whole blood by density gradient centrifugation using Histopaque^®^-1077 (Sigma-Aldrich, St. Louis, Missouri, USA) as density gradient medium. Five millilitres of plasma per donor were preserved for cell culture medium preparation. The PBMCs were then purified by washing them twice with PBS.

### 2.4 Antiviral activity assays

The virus yield reduction assay was conducted at Viroclinics Biosciences BV (Rotterdam, the Netherlands) following the company’s standard operating procedures (SOPs). In brief, Vero E6 cells (for SARS-CoV-2) or Vero cells (for SARS-CoV-1) were seeded at a density of 1.5x10^4^ cells in 96-well plates the day before infection and inoculated with 0.001 MOI of the appropriate virus in the absence (virus control wells) and presence of 100, 75, 50, 25, 12.5, 6.25, 3.125, 1.5625, 0.78125, 0.390625 or 0 µM zapnometinib. The 96-well plates were incubated for 24 and 48 h at 37°C. The supernatants were harvested after the incubation, and the viral titer was determined in four replicates by TrueBlue Immunostaining and spot counting using a CTL Immunospot Image Analyzer. The viral titer measured in the presence of each concentration of zapnometinib was utilized to determine the 50% effective concentration (EC_50_) using a previously described method (45). A cytotoxicity run was conducted concurrently using a lactate dehydrogenase (LDH) release assay as described in supplementary.

For the virus yield reduction assay of SARS-CoV-2 Omicron (B.1.1.592), Calu-3 cells were seeded at a density of 3x10^5^ cells in 24-well plates two days before infection and inoculated with 0.001 MOI of SARS-CoV-2 Omicron (B.1.1.592) for 1 h. Subsequently, the inoculum was completely removed, and the cells were treated with 100, 75, 62.5, 50, 25, 12.5, or 0 µM zapnometinib. Plates were incubated for 24 h at 37°C. The supernatants were harvested after the incubation, and the viral titer was determined using a real time one-step multiplex RT-qPCR method. Briefly, viral nucleic acid extraction was performed using a QIAamp Viral RNA Mini Kit (QIAGEN, Hilden, Germany) following the manufacturer’s instructions. A TaqMan-based RT-qPCR assay was carried out using QuantiNova Pathogen kit (QIAGEN, Hilden, Germany) with specific primers targeting the SARS-CoV-2 N gene (**Supplementary Table 1**). The viral genome copies were determined based on a standard curve prepared with tenfold serial dilutions of the target gene. For QC, nuclease-free water was included in each set of extractions as a negative control to monitor any possible cross-contamination. Moreover, an artificial exogenous QuantiNova RNA internal control (QIAGEN, Hilden, Germany) was included during the nucleic acid purification to monitor the RNA purification efficiency and the RT-PCR amplification. EC_50_ values were calculated based on the measured viral titers normalized to the solvent control (1% DMSO).

The virus yield reduction assay of HCoV-229E and HCoV-OC43 was performed on MRC-5 cells seeded at 1.5x10^5^ cells per well in 24-well plates 24 h prior infection with HCoV-229E (MOI 0.001) or HCoV-OC43 (MOI 0.1) for 1 h. The inoculum was completely removed, and the cells were treated with different concentrations of zapnometinib (50 µM to 0.006 µM) and a solvent control in Iscove’s Modified Dulbecco’s Medium (IMDM) containing 2% FBS, 1% Penicillin/Streptomycin at 34°C, 5% CO_2_ for 24 h (HCoV-OC43) or 48 h (HCoV-229E). Supernatants were harvested and the viral titer was determined by a real-time one-step multiplex RT-qPCR using specific primers targeting the N and NS2 genes for HCoV-229E and HCoV-OC43 respectively (**Supplementary Table 1**) EC_50_ values were calculated based on the measured viral titers normalized to the solvent control (1% DMSO) and using the “log(inhibitor) vs. response - Variable slope (four parameters)” analysis in GraphPad Prism v9.

### 2.5 Drug administration in hamsters

Zapnometinib was synthesized by Chemcon GmbH (Freiburg, Germany). Before administration to Syrian hamsters, it was freshly dissolved in dimethyl sulfoxide (DMSO) (Carl Roth GmbH, Karlsruhe, Germany) and then further dissolved in 15% Kolliphor EL (Sigma-Aldrich, Germany) and 80% phosphate-buffered saline (PBS) (Gibco, Life Technology Europe B.V., Bleiswijk, the Netherlands). Kolliphor EL is used to emulsify and solubilize oils and other water in-soluble substances, it has been used as a pharmaceutical solvent for numerous drugs and has proven in previous *in vivo* studies with zapnometinib. Zapnometinib was administered orally at the indicated doses in a volume of 500 μL/150 g body weight using a gavage needle.

### 2.6 Pharmacokinetics

Drug administration and blood sampling in Syrian hamsters were performed by Viroclinics Xplore (Schaijk, the Netherlands) and are described in the sections “Drug administration” and “Sampling after inoculation”. Specifically, at different time points (0, 0.5, 1, 2, 4, 8, 12, and 24 h) after the administration of a single dose of zapnometinib (15, 30, or 60 mg/kg per oral (p.o.)), ~200 µL blood was collected retro-orbitally from three animals per time point per dose and alternating animals between time points (except at 24 h, n = 6 per group) under isoflurane anesthesia using a heparinized capillary tube containing a clot activator (Microvette 500 Z-Gel, Sarstedt). Serum was collected by centrifuging the tubes at 4000 x g for 10 min, aliquoted, and stored at < -70°C until pharmacokinetic analysis. Daily clinical observations included monitoring of signs such as ruffled fur, hunched back posture, accelerated breathing and lethargy, and the body weight was monitored before and 24 h post administration.

Bioanalysis and pharmacokinetic evaluation were performed at Prolytic GmbH (Frankfurt, Germany). High-affinity liquid chromatography tandem mass spectrometry (HPLC-MS/MS) was employed to determine the concentration of zapnometinib in hamster serum in accordance with the company’s SOPs. During the analytical phase, two batches were evaluated. The acceptance criteria for the calibration standards and quality control (QC) samples were fulfilled for both batches as per the company’s SOPs. The calibration curve was in the range of 10–10000 ng/mL for zapnometinib and showed acceptable linearity over the calibration range. In total, 81 study test samples were analyzed. The validity of the method during the analysis of the test samples was ensured by assaying QC samples with known concentrations of zapnometinib; zapnometinib 13C6 was used as the internal standard. The lower limit of quantification (LLOQ) of the method was determined to be 10 ng/mL for zapnometinib in undiluted serum. All serum concentrations falling below LLOQ were assigned a value of 0. ANALYST software was used to calculate the concentrations of the test and QC samples based on the corresponding calibration curves. The obtained mean serum concentrations were used for noncompartmental pharmacokinetic evaluation with Phoenix^®^ WinNonLin software (Pharsight Corporation, Mountain View, CA, USA; version 8.1).

### 2.7 Efficacy study in the Syrian hamster infection model

On day 0, all animals (n=6 per group) were infected intranasally with SARS-CoV-2 (1x10^3^ median tissue culture infectious dose (TCID_50_) BetaCoV/Munich/BavPat1/2020 (grown and titered at Viroclinics) in a total volume of 0.1 mL diluted in PBS (Gibco, Paisley, UK) and divided between the nostrils. Zapnometinib (100 mg/kg) was administered p.o. (*per os*; oral administration) at either +4 h or +24 h post-infection (p.i.). Subsequently, all animals in both groups received a daily dose of 75 mg/kg zapnometinib. The animals in the control group received vehicle +4 h p.i. and once daily thereafter. All animals were euthanized by exsanguination under 3% isoflurane anesthesia on day 4 post-infection (dpi). All individuals conducting clinical observations and laboratory analyses requiring data interpretation were blinded before the conclusion of the study.

### 2.8 Sampling after inoculation

Respiratory tract samples were collected daily during the study from Syrian hamsters infected and treated as described in the in the section “Efficacy study in the Syrian hamster infection model”. Briefly, throat swabs were collected in virus transport medium (Eagles minimal essential medium containing HEPES buffer, Na bicarbonate solution, L-Glutamine, Penicillin, Streptomycin, BSA fraction V and Amphotericin B), and the samples were aliquoted and stored at < -70°C. Serum was also collected as described for pharmacokinetics at 0 dpi and 4 dpi and frozen at < -70°C until further analysis.

Additionally, upon necropsy, lung, nasal turbinate, and trachea samples were collected and stored in 10% formalin for histopathology or frozen at < -70°C for virological analysis. For virological analysis, the tissue samples were weighed, homogenized in infection medium and centrifuged at 3000 xg for 10 min at room temperature before titration.

### 2.9 Determination of viral load

**Detection of replication-competent virus (TCID_50_ assay):** Quadruplicate 10-fold serial dilutions of throat swabs and tissue homogenate samples were used to determine the viral titers in confluent layers of Vero E6 cells. Briefly, serial dilutions of samples were prepared and incubated on Vero E6 monolayers for 1 h at 37°C. Then, the Vero E6 monolayers were washed once with infection medium (DMEM containing L-Glutamine, Penicillin, Streptomycin, Amphotericin B and Fetal Bovine serum) and incubated for 4–6 days at 37°C, after which the plates were scored using WST8 (colorimetric assessment). The optical density (OD) of the wells was read at 450 nm (OD_450_) using a microplate reader and compared to the OD of the positive control wells (non-treated, infected cells), which showed a cytopathic effect (CPE). The viral titer (TCID_50_) per mL or g was calculated using the Spearman-Karber method.

**Detection of viral RNA:** The viral titer in throat swabs and homogenized tissue samples was determined using an RT-qPCR method. Briefly, RNA was isolated with a Roche MagNA Pure 96 instrument using a MagNA Pure 96 DNA and Viral NA Small Volume Kit, and a TaqMan-based RT-qPCR assay was performed with specific primers (**Supplementary Table 1**), as described by Corman et al. (46). The number of viral copies (CP per mL or g) per sample was calculated by using the resulting Ct value of the sample and the slope, intercept, and upper and lower limits of detection of a log_10_ dilution series of a viral stock for which the number of copies per dilution was known.

### 2.10 Gross pathology

At the time of necropsy, gross pathology was performed for each animal. All lung lobes were inspected, and the percentage of affected lung tissue was estimated from the dorsal view. In addition, any other abnormalities observed in other organs during full-body gross pathology were documented. Then, the left lung lobes, trachea, and nasal turbinates were preserved in 10% neutral buffered formalin for histopathology, while the right lung lobes and nasal turbinates were subsequently homogenized for TaqMan-based RT-qPCR and virus titration.

### 2.11 Histopathology

Histopathological analysis of the left lung, trachea and left nasal turbinate was performed for all animals. After fixation with 10% formalin for at least 2 weeks, tissue sections were embedded in paraffin, micro-sectioned to 3 μm on glass slides and stained with hematoxylin and eosin (H&E) for histological examination. The H&E-stained tissue sections were examined by light microscopy, using an Olympus BX45 light microscope with magnification steps of 40x, 100x, 200x, and 1000x, for histopathology scoring, as well as for the presence of any other lesions. SARS-CoV-2-associated lesions in the lungs were semi-quantitatively assessed as follows: presence of alveolar oedema, alveolar haemorrhage, and type II pneumocyte hyperplasia: 0 = no, and 1 = yes as described previously (47).

### 2.12 Candidate biomarker analysis

Serum samples from hamsters collected at pre- infection (0 dpi) and post-infection (4 dpi) and treatment time points were analyzed to evaluate the levels of various candidate safety biomarkers. Serum proteins were enzymatically fragmented through proteolysis, and protein-specific peptides were targeted for quantification of the corresponding protein biomarker levels by immunoaffinity liquid chromatography tandem mass spectrometry (IA-LC-MS/MS) performed as described previously (48, 49). For further details, see **Supplementary Methods**.

### 2.13 Inflammatory ALI mouse model

Mice were anesthetized by intraperitoneal (i.p.) injection of ketamine solution (10 mg/kg). Both the treatment and control groups (n = 4 each) received 5 mg/kg lipopolysaccharide (LPS) (O55:B5, Sigma-Aldrich) in PBS (100 µL per mouse) via i.p. injection. At 1 h post-stimulation, the treatment group received 25 mg/kg zapnometinib via oral gavage, while the control group received vehicle (15% Kolliphor, 5% DMSO in PBS). The mice were euthanized at 6 h post-zapnometinib treatment, and the lungs were preserved in RNAlater (Qiagen, Hilden, Germany).

Lungs were homogenized by a bead beating system (FastPrep™, MP Biomedicals) and the RNA was isolated using RNeasy Plus Universal Midi Kit (Qiagen) according to the manufacturer’s instructions. RNA quality (A260/A280 ratio ≥1.8 and A260/A230 in the range of 2.0-2.2) was determined using a NanoDrop spectrophotometer (Thermo Fisher Scientific), and the isolated RNA was reverse transcribed using an RT^2^ First Strand Kit (Qiagen). Afterward, the generated cDNA was subjected to a real-time RT² Profiler PCR Array using RT² SYBR^®^ Green qPCR Mastermix. For optimal performance, the - RT² Profiler™ Mouse Cytokines & Chemokines arrays (PAMM-150ZR, Qiagen) were customized to include 84 genes and 5 housekeeping genes for data normalization (see **Supplementary Table 4**). For QC, a mouse genomic DNA contamination test, 3 reverse transcription efficiency tests, and 3 PCR array reproducibility tests were included. The data were analyzed using the GeneGlobe portal (Qiagen) according to the ΔΔCt method. The fold change/regulation was calculated, and the threshold value was 2.

### 2.14 Analysis of cytokine and chemokine levels in human PBMCs

2x10^6^ human PBMCs from two donors each were suspended in 1 mL RPMI 1640 medium (Gibco) containing 5% autologous plasma, 1% penicillin/streptomycin (Sigma-Aldrich), and 1% L-glutamine (Sigma-Aldrich) and were treated with 1 μg/mL LPS from *E. coli* O55:B5 (prepared in PBS) and 10 μg/mL zapnometinib (prepared in DMSO and diluted in cell culture medium to a final concentration of 0.1% DMSO) for 6 h at 37°C and 5% CO_2_. The supernatants (cell culture medium) were collected and stored at -20°C until analysis by ELISA. Ready-to-use ELISA kits (Invitrogen, Waltham, Massachusetts, USA) were used according to the manufacturer’s instructions to determine the levels of the following cytokines: IL-1β, IL-6, IL-8, IP-10, MCP-1, MIP-1α, and TNF-α. Briefly, the samples and standards were applied to ELISA plates pre-coated with specific antibodies targeting the cytokines of interest. Biotinylated detection antibodies binding to the cytokines of interest were added to the wells followed by a horseradish peroxidase (HRP)-streptavidin conjugate, which binds to the biotinylated detection antibodies. Unbound cytokines, detection antibodies, and HRP-streptavidin conjugate were removed by washing. Tetramethylbenzidine (TMB) substrate was added, and the color change in the wells served as the readout for the ELISA. Optical density (OD) at 450 nm was measured using a SpectraMAX Plus 384 microplate reader. For IL-1β, IL-8, MCP-1, and TNF-α the OD was also read at a reference wavelength of 620 nm, and for IL-6 at a reference wavelength of 550 nm. For IP-10 and MIP-1α no reference wavelength was used. Cytokine and chemokine concentrations were calculated following the manufacturer’s instructions for interpolating unknown concentrations from a standard curve.

### 2.15 Virology and immunology from the RESPIRE trial

RESPIRE was a global randomized, double-blind, placebo-controlled proof-of-concept Phase 2 trial in adults with moderate-to-severe COVID-19 requiring hospitalization (clinical severity status (CSS) 3 or 4, measured on a 7-point ordinal scale). The study details are described in Rohde et al., 2023 (42). Briefly, patients were randomized 1:1 to oral zapnometinib (900 mg on Day 1 (D1); 600 mg daily on Day 2-6) or matching placebo, on top of standard of care according to local guidelines. Patients, investigators, the study team, and the Sponsor remained blinded to treatment allocation throughout the study.

Viral load: Nasopharyngeal swabs and sputum were collected at time points D1, D3, D5, D8, D11, D15 and D30 after treatment start and the viral load was quantified via RT-qPCR at CheckImmune GmbH, Berlin.

Cytokine/chemokine: Parameters from 24 (12 zapnometinib, 12 placebo) study participants at time points D1, D3, D5, D8, D11, D15 and D30 were analyzed using MesoScale V-Plex Proinflammatory Panel I and Chemokine Panel and whole Blood IL-8 (MSD, Rockville, USA). For standard of care treatment see **Supplementary Table 5**.

Adaptive immune cell parameters from 12 (6 zapnometinib, 6 placebo) study participants at time points D1, D3, D5, D8, D11, D15 and D30 were analyzed using the following panels: activated/exhausted T cells (Duraclone; Beckman Coulter, Krefeld Germany), activated T cells/Tregs (Duraclone; Beckman Coulter, Krefeld Germany), B cell panel (Duraclone; Beckman Coulter, Krefeld Germany). Patients receiving glucocorticoids as standard of care treatment were excluded from the analyses. Experiments were performed by CheckImmune GmbH according to the companies’ SOPs. In short, presence of lymphocytes, T cells and B cells were determined using Flow cytometry analysis. Fresh whole blood from hospitalized SARS-CoV-2 infected patients was stained with the aforementioned DuraClone antibody panels for 15 min at room-temperature and lysed for 15 min at room temperature following the standard staining protocol. Sample acquisition was carried out using the Navios Flow Cytometer (Beckman Coulter). Data analysis was conducted using Kaluza software before being sent to the LIMS system. This procedure is fit for purpose validated under ISO and GCP guidelines.

### 2.16 Statistical analysis

Unless otherwise noted, data were collected in MS Excel, and statistical analyses were performed using GraphPad Prism software v8 or v9 (La Jolla, CA, USA). Statistical details for each experiment are described in the corresponding figure legends. Differences in viral titer, lung injury severity or body weight were analyzed by one-way ANOVA. Differences in the mRNA expression of cytokines were assessed by analyzing replicate data using one-way ANOVA with the Kruskal-Wallis test or using Student’s t-test (unpaired t-test with Welch’s correction). Differences in cytokine expression at the protein level were analyzed using an unpaired t-test with Welch’s correction for normally distributed data; otherwise, the Mann-Whitney test was applied. Differences in biomarkers were evaluated using Brown-Forsythe ANOVA with the Dunnett T3 correction. P values < 0.05 were considered statistically significant.

## 3 Results

### 3.1 Zapnometinib inhibits propagation of coronaviruses *in vitro*

**Figure 1:**
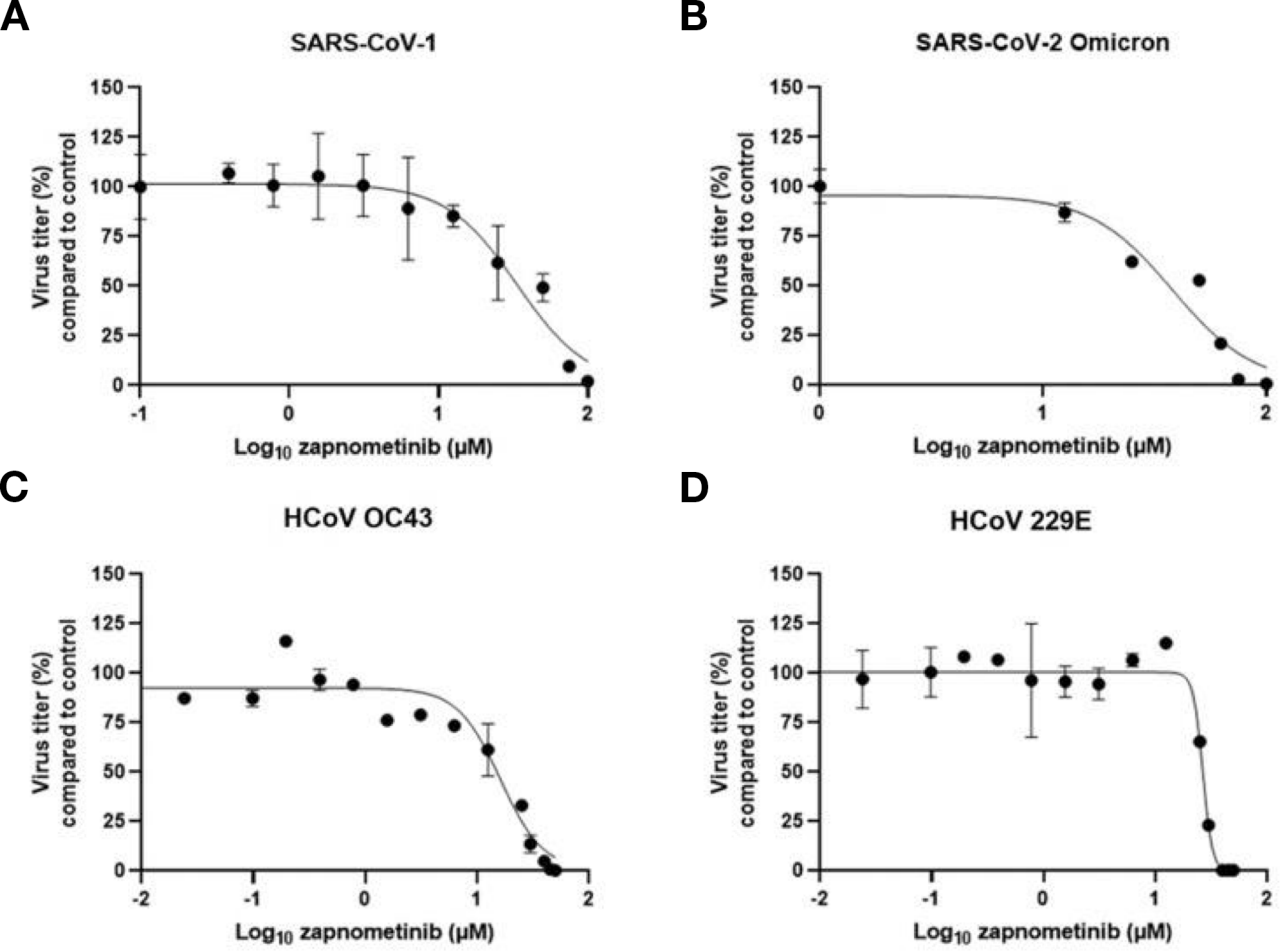
Zapnometinib inhibits various coronaviruses. The antiviral activity of zapnometinib against different Coronaviruses was assessed using a virus yield reduction assay **(B)** SARS-CoV-2 Omicron, **(C)** HCoV OC43 and **(D)** 229E, or virospot reduction assay **(A)** SARS-CoV-1 and the half-maximal effective concentration (EC_50_) was calculated by plotting the viral titer (compared to control) vs. the zapnometinib concentration. The data is shown as mean with standard deviation. n_(SARS-CoV-1)_ = 4; n_(SARS-CoV-2 Omicron)_ = 3; n_(HCoV OC43)_ ≥ 3 (n_(control)_ = 9; n_(12.5, 25, 50 µM)_ = 6; n_(0.006, 0.024, 0.098, 0.195, 0.391, 0.781, 1.563, 3.125, 6.25, 30, 40, 45 µM)_ = 3); n_(HCoV 229E)_ = 3 or 9 (n = 9 for the control). The EC_50_ values are shown in **Table 1**.

We started out with an *in vitro* analysis of the antiviral efficacy of zapnometinib against the SARS-CoV-2 variant “omicron” and other pathogenic human coronaviruses (HCoV OC43, 229E and SARS-CoV-1) using a Virospot or virus yield reduction assay. The viral titer of all tested coronaviruses could be reduced (**Figures 1A–D**). The half-maximal effective concentration (EC_50_) values were in a comparable range, with minor variations attributed to virus-specific assay adjustments, from 16.1 µM (HCoV-OC43) to 37.9 µM (SARS-CoV-2 omicron) for all the viruses tested (**Table 1**). No cytotoxicity was observed in the *in vitro* antiviral assays, as measured by LDH release (**Supplementary Table 2**) and calculated by assessing the 50% cytotoxic concentration (CC_50_) (**Supplementary Figure 1**; **Table 1**). These results confirm and extend the data from our recent findings, demonstrating the antiviral efficacy of zapnometinib against the alpha, beta and delta variants of SARS-CoV-2 in cell culture without inducing cytotoxic effects (36). In summary, the *in vitro* antiviral efficacy of zapnometinib against various coronaviruses has been demonstrated, affirming its broad antiviral therapeutic potential. Moreover, as a host-cell targeting drug, zapnometinib is likely to remain effective against upcoming novel variants or newly emerging zoonotic strains, which is a critical characteristic particularly considering new variants of SARS-CoV-2.

**Table 1:** *In vitro* data. EC_50_ values of zapnometinib for various viruses calculated with a Virospot or virus yield reduction assay; CC_50_ values determined on Calu-3 cells with an LDH cytotoxicity assay after 24 or 48 h zapnometinib treatment.

virus strain	EC_50_ (µM)/(µg/mL)	95% CI (µM)
SARS-CoV-1	33.6/13.8	25.5 - 43.1
SARS-CoV-2 Omicron B1.1.592	37.9/15.5	27.9 - 57.9
HCoV OC43	16.1/6.6	13.6 - 18.6
HCoV 229E	26.6/10.9	25.5 - 27.7
hrs after infection	CC_50_ (µM)/(µg/mL)	95% CI (µM)
24 h	532.1/217.9	422.7 - n/a
48 h	471.0/192.9	439.5 - 496.6

EC_50_, half maximal effective concentration; CI, confidence interval; SARS-CoV, severe acute respiratory syndrome coronavirus; HCoV, human coronavirus; CC_50_, 50% toxic concentration

### 3.2 Pharmacokinetics of zapnometinib in Syrian hamsters

**Figure 2:**
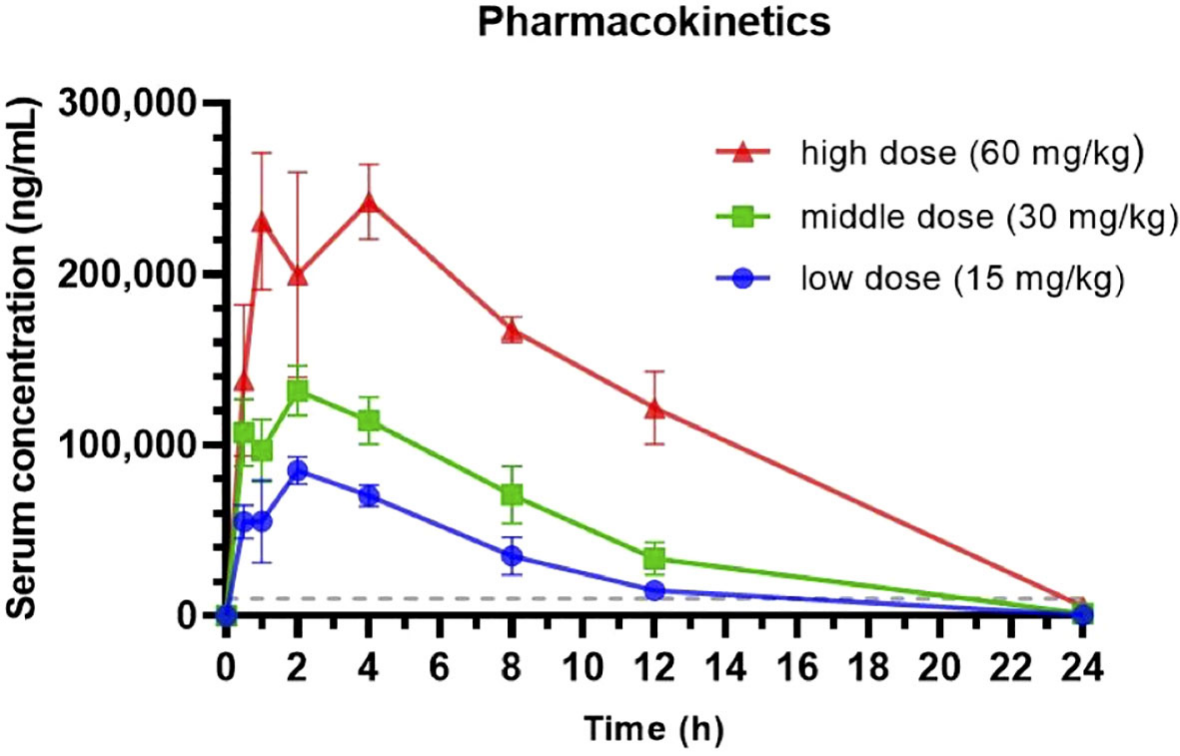
Pharmacokinetic analysis of zapnometinib in Syrian hamsters. Three different doses (15, 30 and 60 mg/kg) of zapnometinib were administered orally to Syrian hamsters (n = 6 per group), and serum was collected 0.5, 1, 2, 4, 8, 12 and 24 h later (alternately from 3 animals per group). The zapnometinib concentration in the serum was determined by HPLC-MS/MS. The data are presented as mean serum concentration ± SD vs. time profiles. The dashed line shows the critical plasma level for MEK inhibition (approx. 10 µg/mL = 24.4 µM).

Next to the *in vitro* efficacy of a drug, we aimed to confirm the therapeutic potential and efficacy in *in vivo* studies. Prior to the *in vivo* efficacy study, we investigated the pharmacokinetics of zapnometinib in Syrian hamsters, a favorable animal model for SARS-CoV-2 infections, to better understand the metabolism of zapnometinib in this animal and to determine an appropriate dose for the subsequent efficacy study. In addition, the tolerability of zapnometinib was assessed by monitoring clinical parameters such as ruffled fur, hunched back posture or lethargy. To investigate dose proportionality, clearance, and safety of zapnometinib in hamsters, single doses of 15, 30 or 60 mg/kg zapnometinib were administered p.o. to groups of 6 animals. Blood samples were collected for analysis after 30 min and after 1, 2, 4, 8, 12, and 24 h from 3 alternating animals per group. The serum concentration-time curves for all doses showed a monophasic progression following oral administration. A proportional increase was observed until the maximum observed plasma concentration (C_max_) which was reached after 2 h for the 15 and 30 mg/kg doses and after 4 h for the 60 mg/kg dose (**Figure 2**). Moreover, the area under the concentration-time curve (AUC) and the C_max_ values increased proportionally with the dose. The apparent terminal half-life was comparable for all dose groups, ranging from of 2 to 3 h (**Table 2**). Notably, at the highest dose (60 mg/kg), the concentration of zapnometinib after 24 h was 5.32 µg/mL (13 µM) (**Table 2**; **Figure 2**). Based on previous work in our laboratory, 10 µg/mL (24.4 µM) is required for 50% MEK inhibition (40). Thus, higher doses are required to attain a serum concentration exceeding 10 µg/mL at 24 h, ensuring effective and consistent MEK inhibition. Additionally, all doses were well tolerated, and no adverse events were reported following zapnometinib treatment. Therefore, based on the current data, a loading dose of 100 mg/kg followed by a daily dose of 75 mg/kg was employed for the efficacy study in the Syrian hamster infection model. This regimen was equivalent to the human dosing used in the phase 2 clinical trial, which involved a higher initial loading dose of 900 mg on day 1, followed by daily dosing with 600 mg zapnometinib. This dosing regimen ensures that the drug concentration remains above 10 µg/mL (24.4 µM) throughout the entire treatment period (40, 42).

**Table 2:** Summary of pharmacokinetic parameters per dose group for zapnometinib in Syrian hamsters.

Group	Dose (mg/kg)	C_max_ (ng/mL)/(µM)	T_max_ (h)	AUC (h*ng/mL)	T1/2 (h)	C_(24 h)_ (ng/µL)/(µM)
1 (n = 6)	60	242434/99.2	4	2970884	3.07	5319.1/2.18
2 (n = 6)	30	131720/53.9	2	1231900	2.75	1347.23/0.55
3 (n = 6)	15	85109/34.8	2	672365	2.74	636.91/0.26

C_max_, maximum observed plasma concentration; T_max_, time to maximum observed plasma concentration; AUC, area under the concentration-time curve; T1/2, terminal half-life; C_(24 h),_ mean concentration at 24 h

### 3.2 Efficacy of zapnometinib in the Syrian hamster infection model

**Figure 3:**
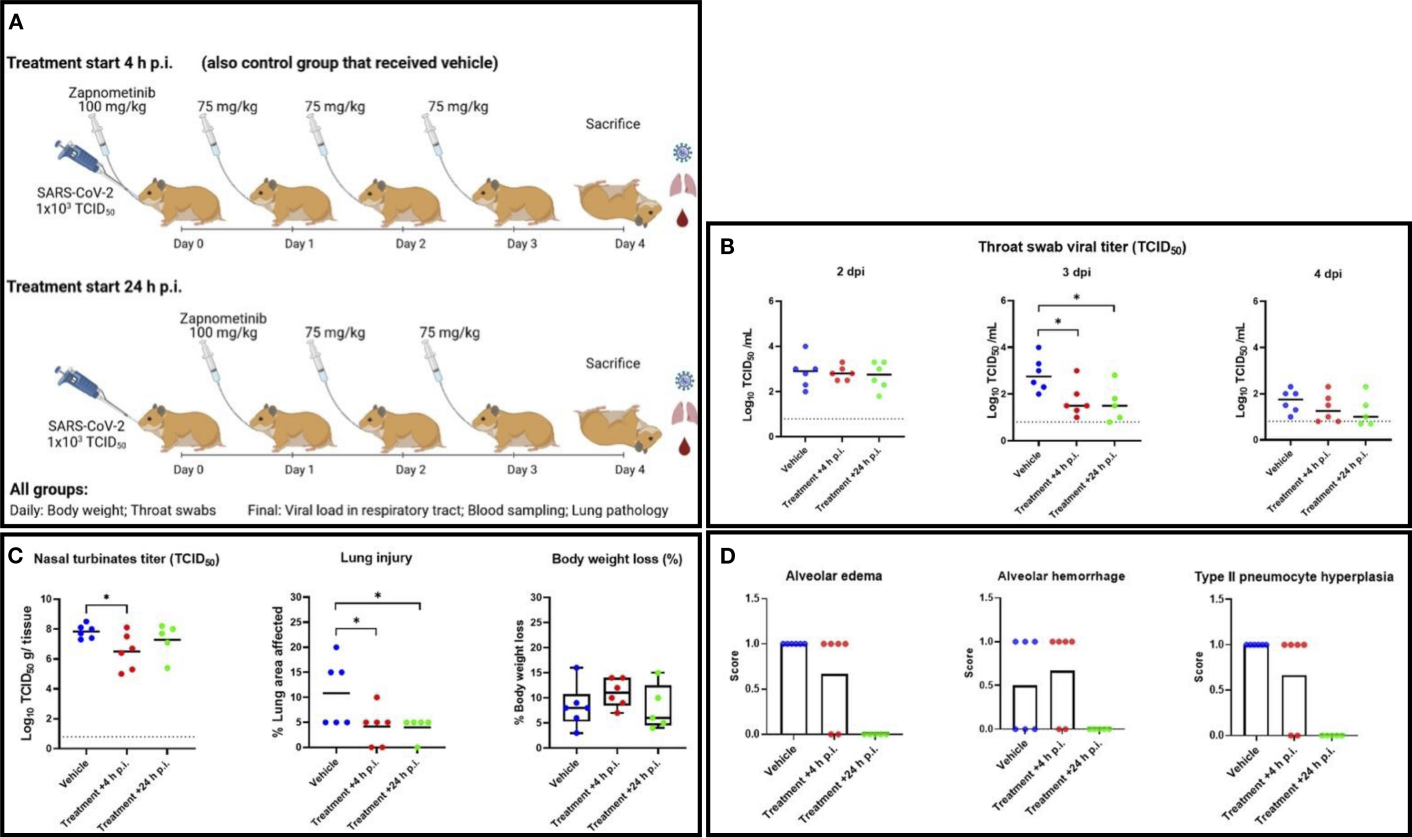
Study of the efficacy of zapnometinib against SARS-CoV-2 in a Syrian hamster infection model. **(A)** Study schedule. On day 0, all animals were infected intranasally with 1x10^3^ TCID_50_ SARS-CoV-2. Zapnometinib (100 mg/kg) was given beginning +4 h p.i. (+ control group that received vehicle) or +24 h p.i. Thereafter, both groups were treated with 75 mg/kg zapnometinib once daily and body weight was measured, and throat swabs were taken daily until sacrifice at 4 dpi. Nasal turbinate tissues were subsequently homogenized and subjected to virus titration. Blood was collected for further analysis, the lungs were inspected and collected for histopathological examination. The study schedule was created on biorender.com. **(B)** Infectious viral titers in throat swabs collected at 2 (left), 3 (middle) and 4 (right) dpi expressed as log_10_ TCID_50_ of control (vehicle-treated) and zapnometinib-treated SARS-CoV-2-infected hamsters. **(C)** left: Infectious viral loads in nasal turbinates collected from control (vehicle-treated) and zapnometinib-treated SARS-CoV-2-infected hamsters at 4 dpi expressed as log_10_ TCID_50_ per g nasal turbinate tissue analysed via TCID_50_ assay. The dashed line represents the lower limit of detection (0.8 log_10_ TCID_50_/mL). **(C)** middle: Percentage of lung area with pathological signs in control (vehicle-treated) and zapnometinib-treated SARS-CoV-2-infected hamsters at 4 dpi. **(C)** right: Weight loss at 4 dpi as a percentage of the body weight measured at the time of infection. Individual data (dots) and median values (horizontal lines) are presented. The whiskers represent the min. and max. values. **(D)** Lung slides were examined and scored for the presence of alveolar edema (left), alveolar haemorrhage (middle) and type II pneumocyte hyperplasia (right). Mean values are presented as percent distribution. All data are from a single experiment. The number of animals per group was as follows: n_(Vehicle)_ = 6; n_(Treatment +4 h p.i.)_ = 6; n_(Treatment +24 h p.i.)_ = 5. The data were analysed by one-way ANOVA. *p < 0.*05*.

An *in vivo* study was designed to investigate the efficacy of zapnometinib administered p.o. in SARS-CoV-2-infected hamsters. In this efficacy study, all animals were challenged with 1x10^3^ TCID_50_ SARS-CoV-2 via intranasal administration, which induces both high levels of viral replication in the respiratory tract and significant histopathological changes in the lungs (50). Control animals were treated with vehicle (5% DMSO, 15% Kolliphor in PBS) beginning at +4 h p.i., while the experimental animals were treated with the following regimen: one loading dose of 100 mg/kg zapnometinib (+4 or +24 h p.i.) followed by 75 mg/kg zapnometinib once per day until the day of sacrifice (4 dpi). During the challenge phase, the animals were weighed, clinical observations were monitored following treatment and infection, and throat swabs were taken daily (**Figure 3A**). At 4 dpi, all animals were euthanized for virological and pathological analyses.

For the analysis of viral titers, throat swabs were titered to detect replication-competent virus. Additionally, they were analyzed using TaqMan-based RT-qPCR to detect viral RNA and determine the viral copy number. At 2 dpi, the viral load in the throat swabs was comparable among all three treatment groups (**Figure 3B**). At 3 dpi, the TCID_50_ assay indicated that zapnometinib treatment in both treatment groups resulted in a significant reduction in the amount of replication-competent virus in the throat swabs, with reductions of 1.1 log_10_ (*p = 0.0134*) and 1.3 log_10_ (*p = 0.0191*) noted in the groups treated at +4 h p.i. and +24 h p.i., respectively. These results were supported by the RT-qPCR data, which demonstrated viral titer reductions (number of viral copies) of 1.1 log_10_ (*p = 0.0089*) and 1.2-log_10_ (*p = 0.0090*) in animals treated at +4 h p.i. and +24 h p.i., respectively, compared to control animals (**Supplementary Figure 2A**). At 4 dpi, the mean viral titer was still lower in the throat swabs of drug-treated animals, although no statistically significant differences were discernible (**Figure 3B**). Nevertheless, in the drug-treated animals, a clear decrease in titer over time (2 dpi, 3 dpi, 4 dpi) was observed, more pronounced than in the control animals.

A similar pattern was observed for the infectious viral load in the nasal turbinates at 4 dpi. Treatment starting at +4 h p.i. significantly reduced the infectious viral titer by 1.3 log_10_ (*p = 0.0335*) (**Figure 3C**). These data were further confirmed by RT-qPCR, which showed a reduction of 1.1 log_10_ (*p = 0.0145*) when treatment was started at +4 h p.i. (**Supplementary Figure 2B**). Similar results were obtained when treatment was started at +24 h p.i. showing a trend toward lower mean viral titers in the nasal turbinates at 4 dpi, although the differences did not reach statistical significance (**Figure 3C**; **Supplementary Figure 2B**).

**Figure 4:**
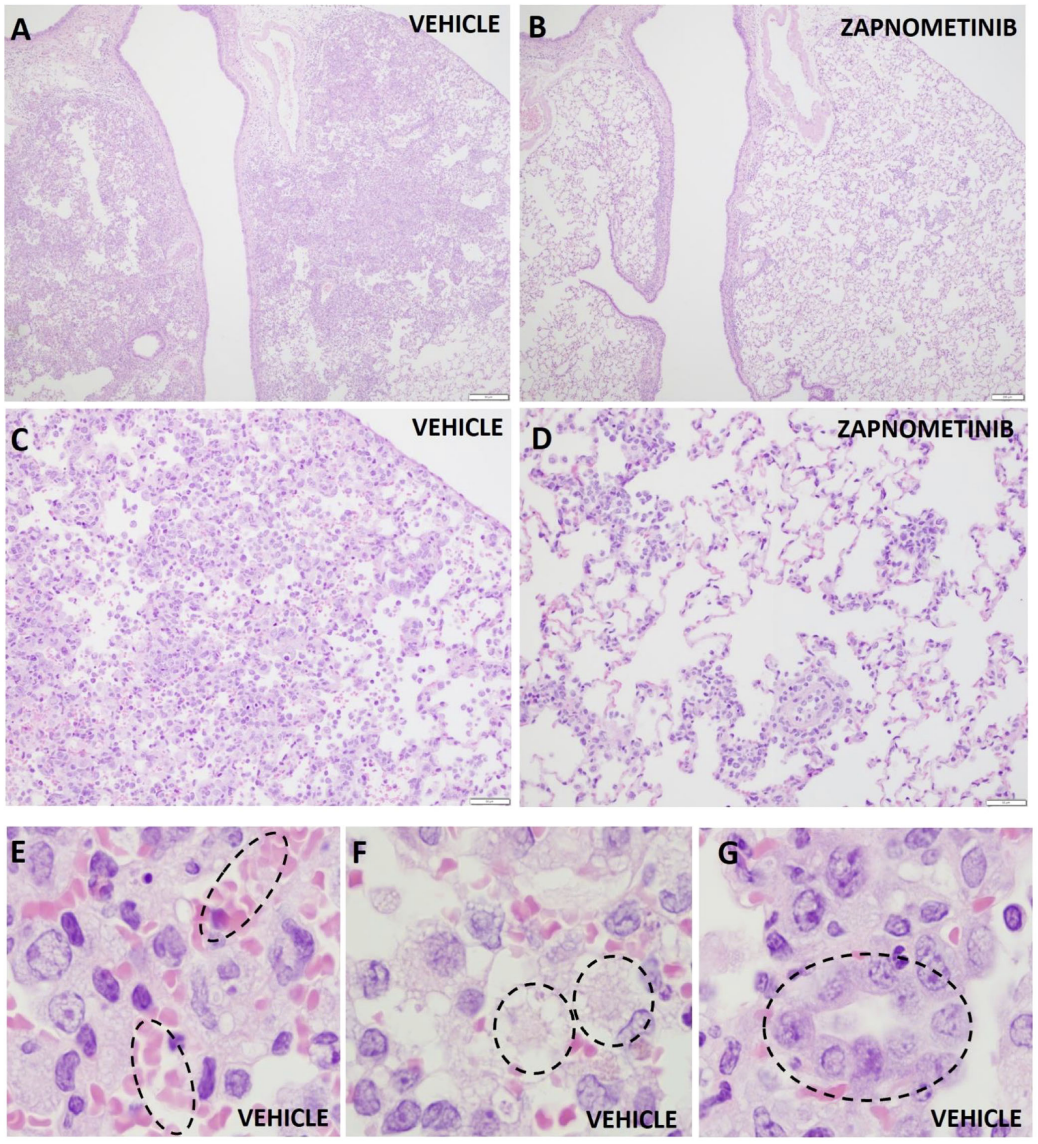
Histopathologic changes in the lungs of hamsters infected with SARS-CoV-2. Representative histopathologic changes at 4 dpi in the lungs of hamsters infected with SARS-CoV-2 and treated with zapnometinib beginning at +24 h p.i. **(B, D)** or treated with the vehicle only **(A, C, E, F, G)**. Haemorrhage **(E)**, edema **(F)** and type II pneumocyte hyperplasia **(G)** were observed in the vehicle only animals (shown in dashed circles). Hematoxylin and eosin staining; 40x magnification **(A, B)**, 200x magnification **(C, D)** and 1000x **(E–G)**.

To gain further insight into how treatment affects disease progression beside its influence on viral load, the lung lesions of the infected animals were evaluated by gross pathology at 4 dpi, and the percentage of affected lung area was estimated. The percentage of lung injury in lung tissue was significantly reduced in both treated groups (*p = 0.0282* for treatment beginning at +4 h p.i., *p = 0.0314* for treatment beginning at +24 h p.i.) compared to the vehicle-treated group, indicating a beneficial effect of zapnometinib treatment (**Figure 3C**). Histopathological evaluation of the lungs revealed that pulmonary changes were mostly mild in all treatment groups. However, inflammation, as indicated by the number of inflammatory cells infiltrating the tissue, consolidation of the tissue, and decreased air containing space in the tissue, was less severe in the zapnometinib-treated groups than in the vehicle-treated control group (**Figure 4**). Hamsters treated with the vehicle showed alveolitis characterized by numerous inflammatory cells, including macrophages and neutrophils in the alveolar septa and lumina, haemorrhage and oedema in the alveolar lumina and type II pneumocyte hyperplasia. In contrast, hamsters treated with zapnometinib exhibited reduced alveolitis with less cellular inflammatory infiltrate containing fewer macrophages and neutrophils and no haemorrhage, oedema, or type II pneumocyte hyperplasia (**Figure 3C**). Comparing the lungs of all treatment groups revealed that alveolar edema, seen as intraluminal proteinaceous fluid, was less frequent in animals treated beginning at +4 h p.i. than in control animals and absent in animals treated beginning at +24 h p.i. Additionally, intra-alveolar haemorrhage, suggestive of epithelial damage and observed as extravasated erythrocytes, was comparable between animals treated beginning at +4 h p.i. and vehicle-treated animals, whereas it was absent in animals treated beginning at +24 h p.i. (**Figure 3C**). The alveolar epithelium showed variable scoring of hyperplasia among the different treatment groups (type II hyperplasia), which is related to regeneration and is seen as plump cuboidal epithelium in the place of the usual flatter alveolar epithelium. Animals treated beginning at +4 h p.i. were less affected, and no hyperplasia was observed in animals treated beginning at +24 h p.i. (**Figure 3C**).

Significant disparities in outcomes between the two treatment regimens were not observed; however, initiating treatment earlier (+4 h p.i.) seemed to enhance the antiviral efficacy of zapnometinib in the nasal turbinates (**Figure 3C**). Conversely, commencing treatment later (+24 h p.i.) exhibited slightly superior effects on certain markers of inflammation in the lungs.

In general, treatment was well tolerated in both groups, as no significant differences in weight were observed among all groups; mean body weight loss was higher than 5% and comparable among all groups (ranging from 8-11%) (**Figure 3C**). In addition, no treatment-related adverse effects were observed in the zapnometinib-treated groups compared to the vehicle-treated group. Taken together, these results demonstrate that zapnometinib treatment is well tolerated in the Syrian hamster model and can exert beneficial effects depending on the timing of administration—significantly reducing viral load in the respiratory tract when initiated early (+4 h p.i.) or alleviating lung pathology when initiated later (+24 h p.i.).

### 3.3 Levels of drug safety biomarkers

**Figure 5:**
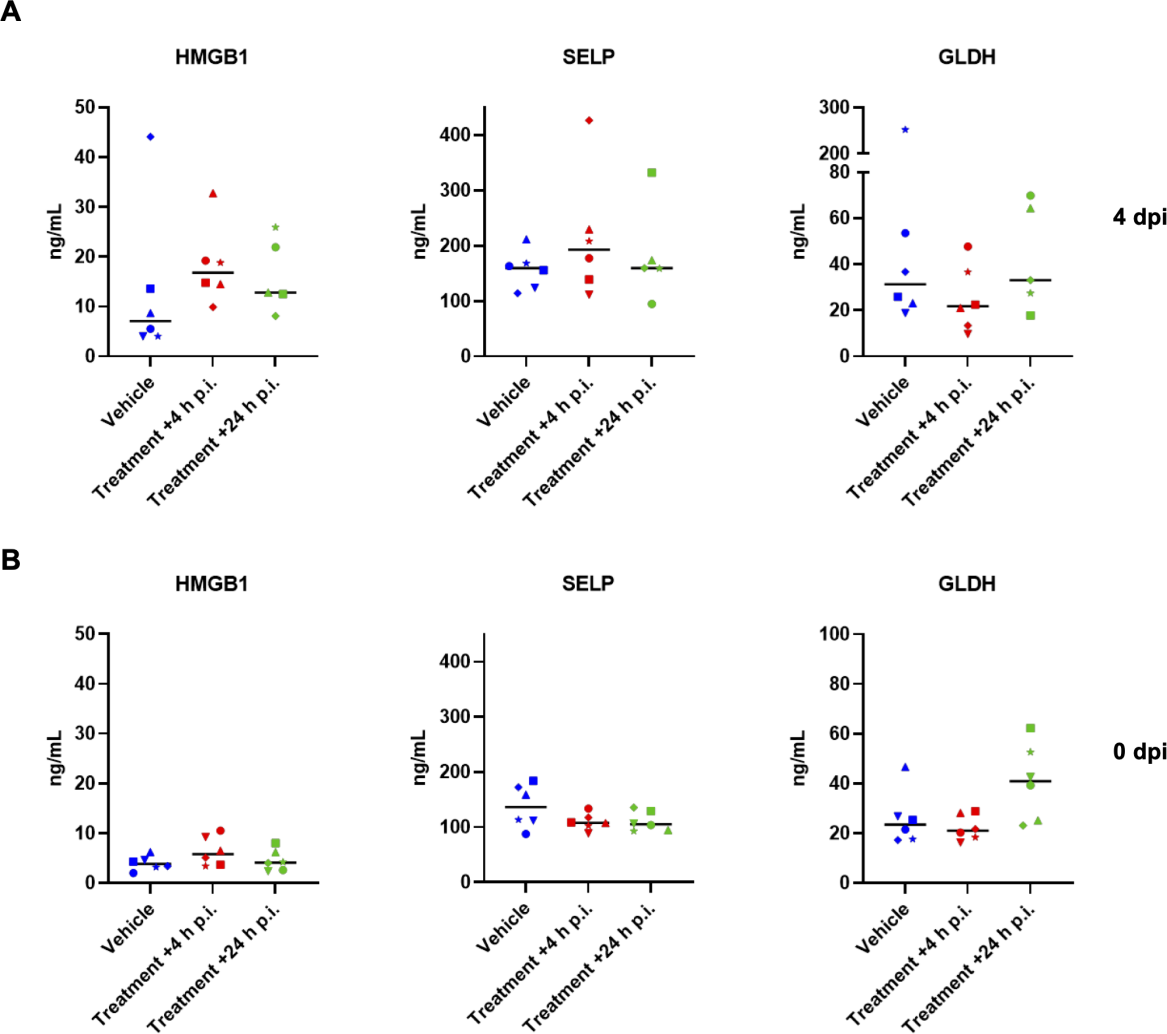
Candidate safety biomarker analysis. The levels of candidate biomarkers in serum samples from hamsters collected **(A)**, at 4 dpi after infection and zapnometinib treatment and **(B)**, before infection with SARS-CoV-2 (0 dpi, prior to infection and zapnometinib treatment) and were analysed by IA-LC-MS/MS. High mobility group box 1 protein (HMGB1, an indicator of necrosis), glutamate dehydrogenase (GLDH, an indicator of liver damage), and p-selectin (SELP, an indicator of endothelial damage) levels in the zapnometinib-treated and vehicle-treated groups are shown. The treatment conditions described below the graphs refer to the treatment that the animals received after infection. Individual data (different symbols) and median values are presented. The number of animals per group was as follows: n_(Vehicle)_ = 6; n_(Treatment +4 h p.i.)_ = 6; n_(Treatment +24 h p.i.)_ = 5. The data were analysed by one-way ANOVA. P values < 0.05 were considered statistically significant.

To further monitor possible drug-related adverse effects in the hamster study, serum samples collected at baseline (0 dpi) and at 4 dpi following infection and treatment with zapnometinib or vehicle. These samples were analyzed for candidate protein biomarkers indicative of organ injury. Various candidate biomarkers were analyzed to evaluate drug-induced vascular injury (DIVI) (51) and drug-induced liver injury (DILI) (51, 52). The levels of high mobility group box 1 (HMGB1; an indicator of necrosis) (53, 54) and glutamate dehydrogenase (GLDH; an indicator of liver damage) (55) were measured to assess liver injury (DILI), and p-selectin (SELP) was used as an early indicator of endothelial damage to blood vessels (DIVI). There were no prominent changes in the levels of the examined biomarkers before and after infection and treatment of the animals, and the levels always remained comparable among all groups (**Figure 5**). When comparing the values from day 0 to day 4, it can also be observed that there was no significant increase in the biomarkers due to the infection. Collectively, these results provide further evidence that the selected treatment regimen (100 mg/kg zapnometinib followed by 75 mg/kg beginning at +4 or +24 h p.i.) was not toxic to hamsters.

### 3.4 Effect on cytokines and chemokines in preclinical models

**Figure 6:**
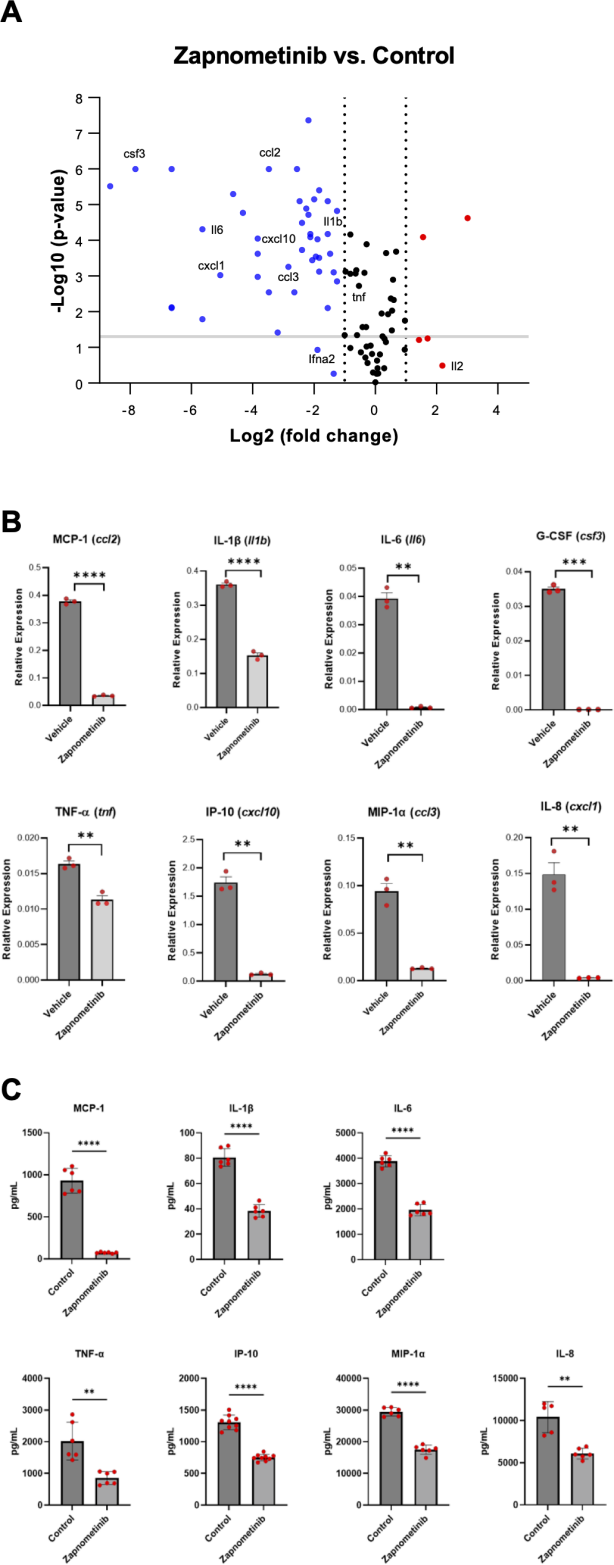
Levels of cytokines and chemokines associated with COVID-19 after zapnometinib treatment. mRNA expression of different cytokines and chemokines in acute lung injury (ALI) model mice after LPS stimulation and zapnometinib treatment 1h post stimulation (control group received vehicle =1% DMSO in PBS). The mRNA levels were determined at 6 h post treatment by an RT^2^ profiler PCR array. **(A)** Volcano plot shows gene expression changes. Representative genes mainly associated with COVID-19 are highlighted. Red: upregulation, blue: downregulation, black: no change in gene expression (regardless of statistical significance). The log_2_ of the fold change in gene expression is plotted on the x-axis, and statistical significance is plotted on the y-axis. The two dashed vertical lines indicate the fold change threshold, while the horizontal line indicates the p value threshold. Genes represented by data points in the far upper left (downregulated) and far upper right (upregulated) sections met the fold change and p value thresholds, while the genes represented by the data points between the two vertical lines showed no change in gene expression. **(B)** Representative expression. Mean + SD and individual values of n = 3 technical replicates are shown, gene names in brackets. The data were analysed using the ΔΔCt method and normalized to the mean expression levels of 5 housekeeping genes. The P values for the difference in replicate (-DeltaCt) expression data for each gene between the control and treatment groups were calculated by unpaired t-test with Welch’s correction. **p < 0.*01*, ***p < 0.*001*, ****p < 0.*0001*. **(C)** Reduction in cytokine expression after zapnometinib treatment in LPS-stimulated human PBMCs from two healthy donors. Cells were simultaneously stimulated with 1 µg/mL LPS and treated with 10 µg/mL zapnometinib for 6 h. The expression of cytokines in the supernatants was analysed by ELISA. The data are presented as the mean ± SD of technical replicates. n_(IL-1β; IL-6; MCP-1; MIP-1α; TNF-α)_ = 6; n_(IP-10)_ = 9; n_(IL-8 control)_ = 5; n_(IL-8 zapnometinib-treated)_ = 6. The differences in the levels of all cytokines of interest between the control and zapnometinib-treated groups were analysed with unpaired t-test with Welch’s correction. **p < 0.*01;* ****p < 0.*0001.* Results from the second donor are shown in **Supplementary Figure 4**.

In addition to demonstrating antiviral efficacy, we also investigated the potential immunomodulatory properties of zapnometinib, focusing on cytokine and chemokine expression. However, in the SARS-CoV-2-challenged Syrian hamster model, the levels of cytokines and chemokines increase only marginally. Therefore, the model is inappropriate for assessing alterations in cytokine and chemokine levels (56). We confirmed this observation in serum samples from the hamsters obtained from the efficacy study using ELISA, with no major differences between the groups after infection and treatment at 4 dpi (**Supplementary Figure 3A**) and in lung tissue samples analyzed by RT-qPCR from the same animals at 4 dpi (**Supplementary Figure 3B**). As the effects investigated in the hamster model do not clearly show the immunomodulatory component of zapnometinib, we aimed to demonstrate a direct anti-inflammatory effect in the absence of a dynamic stimulus such as virus infection. We used an LPS-induced acute lung injury (ALI) mouse model to simulate a pathogen-induced hyperinflammatory disease with high severity. Using this virus-independent model, we could demonstrate the direct effect of zapnometinib on cytokine and chemokine expression. Mice were challenged by i.p. injection of 5 mg/kg LPS and then treated with 25 mg/kg zapnometinib 1 h post-stimulation. Alterations in cytokine and chemokine gene expression patterns were evaluated by comparing them with untreated control mice using an RT^2^ profiler PCR array. As depicted in the volcano plot, the expression of most of the 84 genes encoding key secreted proteins central to inflammation, such as IL-1β (*Il1b*), IL-6, MCP-1 (*ccl2*), IL-8 (*cxcl1*), IP-10 (*cxcl10*) and MIP-1α (*ccl3*), was downregulated after zapnometinib treatment compared to control treatment (**Figure 6A**). Importantly, the levels of the *Ifna2* gene, encoding for IFN-α, a key regulator of the innate antiviral immune response, were not significantly downregulated (*p = 0.118*). The expression of various important genes known to be elevated in COVID-19, such as *Il6*, *Cxcl1* and *Tnf* (57) was significantly reduced in the lung tissues of zapnometinib-treated mice (**Figure 6B**). These data represent the first *in vivo* confirmation of previous *in vitro* findings that zapnometinib affects the proinflammatory gene expression response while genes of the antiviral type I IFN response remain largely unaffected (36).

In addition to the animal model, we aimed to demonstrate the immunomodulatory properties of zapnometinib in a primary human cell type, specifically freshly isolated human PBMCs. PBMCs obtained from two healthy donors were challenged with 1µg/mL LPS from *E. coli* O55:B5 (prepared in PBS) and treated with 10 μg/mL zapnometinib (prepared in 0.1% DMSO) for 6 h. Cytokine and chemokine expression analysis by ELISA confirmed the anti-inflammatory properties of zapnometinib in human PBMCs. Expression levels of IL-1β, IL-6, IL-8, IP-10, MCP-1, MIP-1α, and TNF-α were significantly reduced upon treatment with zapnometinib for 6 h compared to the untreated control group (**Figure 6C**, **Supplementary Figure 4**). Taken together, these experiments collectively validate the anti-inflammatory properties of zapnometinib both *in vivo* and in primary human blood cells, demonstrating a significant reduction of cytokines and chemokines known to be elevated in COVID-19.

### 3.5 Effect on cytokines and chemokines in the clinical trial

**Figure 7:**
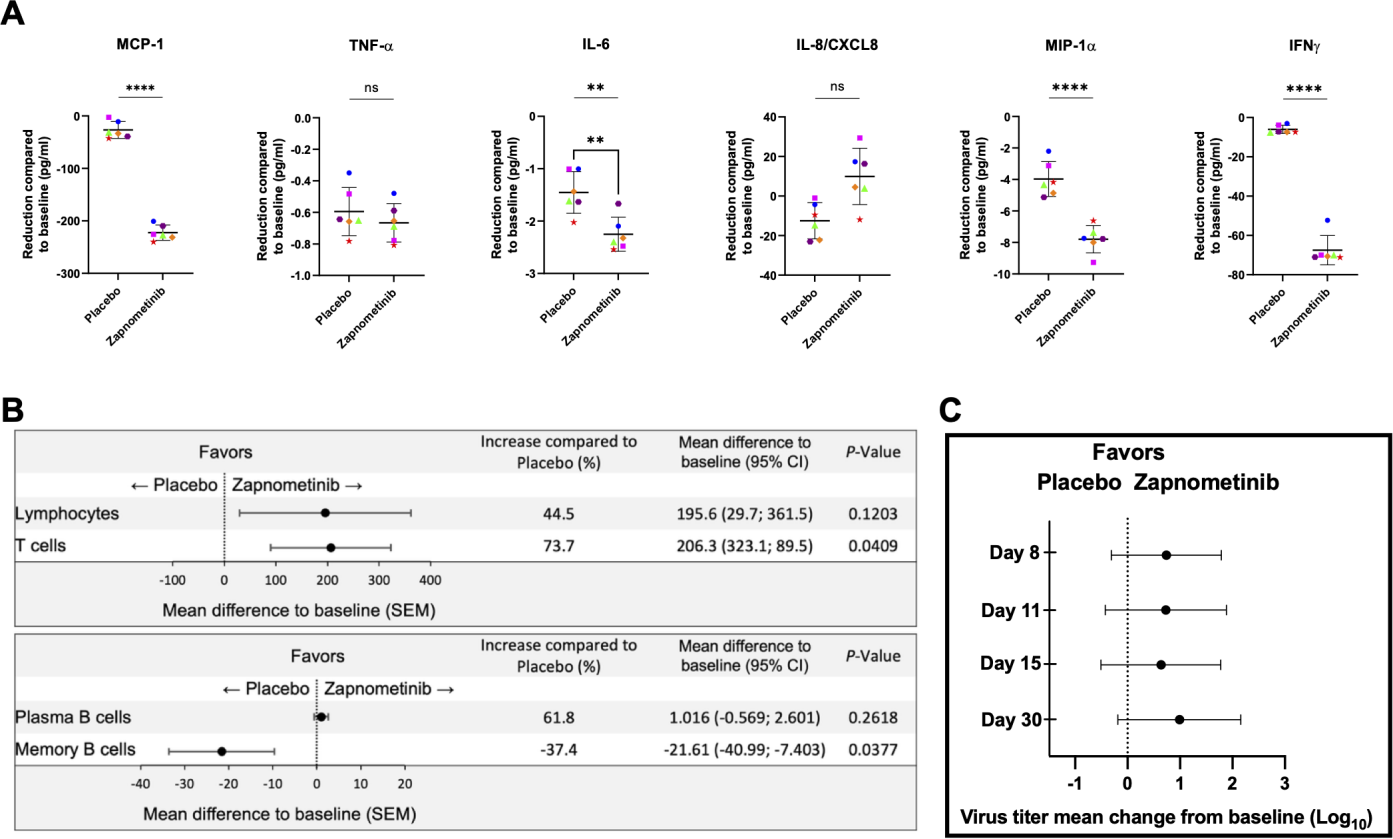
Immunomodulating effects and viral load analysis of patients hospitalized with COVID-19 and treated with either zapnometinib or placebo for 6 days. **(A)** Reduction of cytokine and chemokine expression compared to baseline values. Analysis was performed with blood from 6 patients from each group using Meso Scale V-Plex. All of them received either no standard of care treatment or antiviral treatment. Patients that received glucocorticoids as standard of care were excluded. Analysis was performed with values from the complete observation period (until day 30), horizontal line showing the mean value ± SEM of all days. Colour and symbols represent the mean values of n = 6 patients on different days: • blue circle = day 3, pink square = day 5; green triangle = day 8; orange rhombus = day 11, aubergine-coloured hexagon = day 15, red star = day 30 ▲. Analysis was performed using a one-tailed t-test. **p < 0.*01;* ****p < 0.*0001.* **(B)** Mean difference ± SEM of the number of lymphocytes, T cells, Plasma B cells and Memory B cells compared to baseline in zapnometinib (n = 34) and placebo treated (n = 34) patients. The graphics represent values from the entire study period (D1, D3, D5, D8, D11, D15 and D30). The data were analysed by one-tailed t-test. **(C)** RNA virus titer mean change from baseline over time in SARS-CoV-2 sputum samples from hospitalized COVID-19 patients on D8, D11, D15 and D30. Analysis only includes patients with baseline sputum SARS-CoV-2 RNA titer ≥ 500 copies/ml. Difference of mean change from zapnometinib vs. placebo treatment was given as Log_10_ copies/ml ± SEM, analysed with unpaired t-test with Welch’s correction. Further details of analysis see **Supplementary Table 3**.

To determine whether the reduction in cytokine and chemokine levels following zapnometinib treatment was also observed in hospitalized COVID-19 patients, serum samples from six patients in each group of a randomized, double-blind, placebo-controlled proof-of-concept Phase 2 trial in adults with moderate-to-severe COVID-19 symptoms (clinical severity status CSS 3 or 4; RESPIRE; NCT04776044) were analyzed. Samples were collected at various time points from day 1 to day 30 (D1, D3, D5, D8, D11, D15 and D30) after treatment initiation using a multiplex ELISA system. Despite the absence of a pronounced inflammatory response suggestive of a cytokine storm in the patients, as reflected by their clinical severity scores (CSS3/4), a significant reduction was observed in MCP-1 (*p = <0.0001*), MIP-1α (*p = <0.0001*), and IFNγ (*p = <0.0001*) levels among individuals treated with zapnometinib compared to those in the placebo cohort throughout the duration of the study period (up to day 30). IL-6 levels were significantly reduced (*p* = *0*.*0017*), while TNF-α showed no changes and IL-8/CXCL-8 levels displayed a slight but not statistically significant increase (**Figure 7A**). These results further support the findings from the pre-clinical investigations that zapnometinib treatment leads to a reduction of various pro-inflammatory cytokines and chemokines.

### 3.6 Modulation of adaptive immune response in the clinical trial

In addition to the cytokine storm observed in the hyperinflammatory stage in moderate to severe cases of COVID-19, there is also a decrease in the adaptive immune response. Therefore, we also investigated the impact of zapnometinib on the overall number of lymphocytes, as well as T and B cells, which were quantified in the blood using FACS analysis at the same time points as the cytokine analysis. Elevated levels of lymphocytes, plasma B cells, and T cells were observed in individuals undergoing treatment with zapnometinib (**Figure 7B**), indicating a beneficial immune enhancement that augments the antiviral response following zapnometinib administration. Notably, a substantial 44.5% increase in lymphocyte count was documented during the 30-day observation period post-zapnometinib treatment. For T cells, the increase was even more pronounced (73.7%; *p* = *0*.*0409*). As for Plasma B cells, there was also an increase of 61.8% in favor of zapnometinib, while a reduction was found for memory B cells (-37.4%; *p* = *0*.*0377*) (**Figure 6B**). The difference in favor of zapnometinib was consistent across all observation days. Thus, we demonstrate here a positive immunomodulatory effect of zapnometinib on both the innate and adaptive immune responses.

### 3.7 Antiviral effect of zapnometinib in the clinical trial

Besides the immunomodulatory effect, the antiviral effect of zapnometinib was also investigated in patients participating the above-mentioned clinical trial. Therefore, sputum and nasopharyngeal swabs were collected on days D1, D3, D5, D8, D11, D15, and D30 after treatment initiation, and the viral load was determined by RT-qPCR. In the modified intention-to-treat population, 62 out of 103 patients (60.2%) had quantifiable RNA above 500 copies/ml confirmed in nasopharyngeal samples at baseline, and 74 out of 103 patients (71.8%) had RNA above 500 copies/ml confirmed in sputum samples (**Table 3**). Patients in the study received either antiviral, glucocorticoid or no standard of care treatment, with the presence of alpha, beta, gamma, delta and omicron strains. Zapnometinib treatment was associated with greater reductions from baseline in mean viral load than placebo in sputum samples at days 8, 11, 15 and 30 (**Figure 7C**, **Table 3**). However, at days 3 and 5, there was only a slight reduction in favor of zapnometinib (**Table 3**). In participants infected with non-Omicron variants, Zapnometinib demonstrated a consistent and clinically meaningful reduction in SARS-CoV-2 RNA titers compared to placebo, with a statistically significant difference of +1.57 log_10_ on Day 8 (p=0.0101). Although statistical significance was narrowly missed on Days 11, 15, and 30 (p=0.0551–0.0672), the differences remained above 1 log_10_ at all time points. In nasopharyngeal samples, greater reductions from baseline in mean viral load in favor of zapnometinib were also found, albeit to a lesser extent (**Supplementary Table 3**).

**Table 3:** Mean viral titer and mean change from baseline (Day 0) over time (Day 3 – Day 30) in SARS-CoV-2 RNA titer from sputum samples (all randomized MITT population) from patients of the RESPIRE clinical trial treated either with zapnometinib or placebo D1 - D6.

Sputum											
Visit	Zapnometinib				Placebo				Zapnometinib - Placebo		
	n	Baseline Mean	Mean Change^a^ (SD)		n	Baseline Mean	Mean Change^a^ (SD)		Difference	(95% CI)	p-value
SARS-CoV-2 RNA titer (log10 copies/ml) in participants overall											
Baseline	37	6.85			34	6.46					
Day 3	36	5.79	1.08	(2.08)	34	5.51	0.94	(1.98)	0.14	-0.827 to 1.112	0.3854
Day 5	34	5.40	1.48	(1.98)	31	5.01	1.38	(1.56)	0.10	-0.785 to 0.977	0.4145
Day 8	31	4.04	2.81	(1.98)	27	4.35	2.07	(1.99)	0.74	-0.308 to 1.785	0.0814
Day 11	28	3.39	3.68	(2.18)	25	3.39	2.95	(2.00)	0.73	-0.426 to 1.883	0.1054
Day 15	30	2.98	3.93	(2.19)	4	3.74	3.36	(2.11)	0.56	-0.570 to 1,697	0.1619
Day 30	27	2.26	4.74	(1.81)	24	2.47	3.75	(2.23)	0.97	-0.175 to 2.102	**0.0476b**
SARS-CoV-2 RNA titer non omicron (log10 copies/ml) in participants overall											
Baseline	24	6.75			21	6.42					
Day 3	23	5.80	1.16	(2.25)	21	5.47	0.96	(1.90)	0.21	-1.057 to 1.472	0.3709
Day 5	23	5.60	1.28	(2.09)	18	5.24	1.07	(1.28)	0.21	-0.867 to 1.278	0.3501
Day 8	20	4.24	2.63	(2.26)	15	5.26	1.06	(1.55)	1.57	0.261 to 2.883	**0.0101b**
Day 11	17	3.61	3.62	(2.62)	14	4.05	2.20	(2.17)	1,42	-0.341 to 3.174	0.0551
Day 15	19	3.43	3.59	(2.47)	16	3.74	2.40	(2.08)	1.18	-0.385 to 2.744	0.0672
Day 30	16	2.52	4.61	(1.92)	14	2.61	3.37	(2.16)	1.24	-0.311 to 2.797	0.0561
SARS-CoV-2 RNA titer omicron (log10 copies/ml) in participants overall											
Baseline	13	7.02			13	6.51					
Day 3	13	5.78	0.94	(1.82)	13	5.59	0.92	(2.19)	0.02	-1.608 to 1.656	0.4881
Day 5	11	5.04	1.91	(1.75)	13	4.69	1.82	(1.86)	0.09	-1.442 to 1.618	0.4532
Day 8	11	3.73	3.12	(1.36)	12	3.21	3.32	(3.32)	-0.20	-1.579 to 1.171	0.3801
Day 11	11	3.10	3.77	(1.38)	11	2.57	3.90	(1.31)	-0.13	-1.326 to 1.066	0.4115
Day 15	11	2.31	4.52	(1.56)	12	1.90	4.64	(1.39)	-0.13	-1.410 to 1.162	0.4216
Day 30	11	1.95	4.92	(1.71)	10	2.28	4.34	(2.15)	0.58	-1.222 to 2.380	0.2534

Analysis only includes patients with baseline sputum SARS-CoV-2 RNA titer ≥ 500 copies/ml. Baseline results below this limit were excluded from the analysis.

a Mean change from baseline values are based on the measurements from patients with values at both baseline and at the time point assessed. b p < 0.050.

In summary, the positive immunomodulatory properties, and the antiviral effect of zapnometinib demonstrated in various preclinical studies were confirmed in hospitalized patients with a severe viral infection.

## 4 Discussion

Recent cell culture studies have provided initial evidence that the MEK inhibitor zapnometinib exhibits anti-SARS-CoV-2 activity *in vitro* and downregulates the expression of candidate cytokines, suggesting a potential dual benefit in COVID-19 treatment. The present study aimed to substantiate this dual effect through extended preclinical work and further confirm it with clinical data. We evaluated whether these *in vitro* activities translate into efficacy in an infected organism, thereby assessing whether zapnometinib displays a dual therapeutic effect *in vivo*. Therefore, we analyzed the antiviral efficacy and immunomodulatory impact of zapnometinib using a well-established Syrian hamster SARS-CoV-2 infection model and in other models of hyperinflammatory infectious diseases. This study was a first attempt to demonstrate the efficacy of zapnometinib against SARS-CoV-2 *in vivo*. Further details, such as an optimized treatment regimen, need to be clarified in further studies. Given that *in vitro* activities of antiviral compounds are not always reflected *in vivo*, it is very important that a dual effect of zapnometinib could also be demonstrated *in vivo*. This is evidenced by reduced levels of both viral RNA and infectious particles in hamster throat swabs and nasal turbinates, an ameliorative effect on lung pathology as well as the results of a clinical phase 2 trial with hospitalized COVID-19 patients. The results emphasize the therapeutic potential of zapnometinib against coronavirus infections correlated with hyperinflammatory symptoms.

Zapnometinib, a host-targeted antiviral (HTA) inhibits the cellular Raf/MEK/ERK signaling pathway that is pivotal for replication of various viruses (58). It is important to note that the EC_50_ values observed for zapnometinib *in vitro* (16.1–37.9 μM) are higher than those typically reported for direct-acting antivirals (DAAs). However, zapnometinib as HTA inhibits a cellular signaling pathway essential for viral replication rather than acting directly on viral proteins. This fundamental difference in mode of action means that higher compound concentrations are required *in vitro* to achieve an antiviral effect, because the drug must sufficiently inhibit the host cell factor to disrupt viral replication indirectly. Such, *in vitro* EC_50_ values are therefore not directly comparable to those of DAAs. *In vivo*, the pharmacokinetic–pharmacodynamic relationship is also governed by tissue distribution, target engagement, and the modulation of host immune responses, which cannot be fully captured by cell culture assays. The clinical feasibility of the dosing has been demonstrated in two human phase 1 studies and in the present phase 2 study, where zapnometinib was well tolerated at doses designed to achieve sufficient systemic exposure for MEK inhibition without notable toxicity. This distinction is especially important when comparing zapnometinib to currently approved DAAs. As of February 2024, the DAAs nirmatrelvir, molnupiravir (emergency use in the U.S.) and remdesivir were considered and approved for treatment against COVID-19 by FDA and/or EMA (16, 59–61). In the Syrian hamster model of SARS-CoV-2 infection, zapnometinib showed a significant effect on viral load reduction at 3 dpi (**Figure 3B**) comparable to that of the direct-acting nucleoside analogue molnupiravir (59). Furthermore, zapnometinib treatment resulted in a reduction in lung pathology. Based on the data, there is no clear indication which start of treatment (+4 h p.i. or +24 h p.i.) is ultimately more beneficial. The assessment of the precise contribution of the immunomodulatory component of zapnometinib versus the reduction in viral load to the observed positive impact on lung pathology in the hamster model remains indeterminate. Notably, this model did not include an in-depth analysis of the effects on the adaptive immune response. However, our latest publication involving IAV-infected mice demonstrated that zapnometinib positively influences disease pathogenesis by modulating regulatory T cells, indicating that zapnometinib affects not only the expression of cytokines and chemokines (62). Future investigations are warranted to systematically explore and elucidate the specific mode of action of zapnometinib in this context. Nevertheless, the data from the hamster study could also substantiate the therapeutic success observed in the RESPIRE study (42). We do not anticipate zapnometinib, being a host-targeted antiviral agent, to induce mutagenic effects on the virus. In contrast, concerns have been raised regarding the mutagenic potential of nucleoside analogue antivirals like molnupiravir and, less frequently, remdesivir, which could potentially enhance viral evolution in treating viral diseases (63–65). This concern is not applicable to host-targeted antivirals, as they do not interfere with viral RNA replication. Another major advantage of host-targeted antivirals is their high barrier against the emergence of antiviral resistance. This is because the virus faces difficulty in compensating for the absence of cellular functions targeted by the drug. This was evidenced in a resistance study with zapnometinib compared to various direct-acting SARS-CoV-2 drugs (66). Drug resistance to current DAAs has already been documented, presenting a challenging problem that could potentially be mitigated by HTAs (67, 68). Given the stable nature of viral dependence on cellular factors, zapnometinib’s antiviral activity is likely to remain effective against new and emerging variants. Notably, zapnometinib demonstrated efficacy against a spectrum of coronaviruses, including SARS-CoV-1, SARS-CoV-2 Omicron variant, HCoV-OC43, and HCoV-229E (**Figure 1**), underscoring its broad-spectrum potential. In the phase 2 RESPIRE study, various SARS-CoV-2 variants were identified in the patients (alpha, beta, gamma, delta and omicron). The reduction in propagation observed in both omicron and non-omicron strains after zapnometinib treatment underscores the strong correlation between pre-clinical and clinical data. The time course of viral RNA reduction in the RESPIRE trial also deserves further discussion. The data (**Table 3**) show that reductions in viral load were modest at days 3 and 5 but became more pronounced and consistent from day 8 onwards, persisting through day 30. Since treatment was discontinued after day 7, the later differences cannot be directly attributed to ongoing drug exposure. One possible explanation is that zapnometinib not only reduces viral replication during treatment but also enhances cellular immune responses that may support clearance of infected cells at later stages. At the same time, the contrasting patterns observed between sputum and nasopharyngeal swabs cannot yet be fully explained. While this discrepancy lies beyond the scope of the present manuscript, the findings nevertheless suggest a potential benefit of zapnometinib even when administered beyond the very early phase of infection.

Interestingly, no viral load reduction was observed after molnupiravir treatment in hospitalized COVID-19 patients with a similar CSS as in RESPIRE (69). In a clinical trial involving nirmatrelvir/ritonavir (Paxlovid™) administered to hospitalized patients, no statistically significant variance was observed in the duration of SARS-CoV-2 RNA clearance or mortality rates (70). For high-risk patients, a favorable impact on the severity of COVID-19 is delineated (71). However, even if the two approved medications effectively reduce the incidence of hospitalizations and fatalities in non-hospitalized adults with mild-to-moderate COVID-19 in clinical trials, there remains an absence of a viable treatment option for severely ill patients, the specific patient population for which zapnometinib is intended (72–74). The efficacy of molnupiravir and Paxlovid™ in hospitalized patients appears to be constrained. This represents a critical gap in available treatments that could potentially be addressed by the introduction of zapnometinib.

Understanding cytokine pathways is crucial for developing effective treatments, with ongoing trials exploring anti-inflammatory and antiviral drugs’ potential in combating cytokine storms. Additionally, dietary interventions, such as zinc or vitamins, are being studied to regulate cytokine levels and alleviate COVID-19 symptoms. Zinc’s anti-inflammatory properties are being evaluated in clinical trials to assess its impact on the immune response and disease prognosis. However, careful risk assessment is necessary for immunomodulatory approaches due to the risk of secondary infections or impairment of viral clearance (17–19).

A major emphasis of the study was to explore the immunomodulatory effects of zapnometinib *in vivo*, as a second beneficial activity alongside its antiviral action. As anticipated, no significant difference in cytokine and chemokine expression was observed at 4 dpi in the SARS-CoV-2 infected Syrian hamster, indicating its unsuitability for infection-related cytokine studies (56). Therefore, we opted for an *in vivo* acute lung injury (ALI) model which represents a fulminant hyperinflammatory condition independent of pathogen infection dynamics. While the LPS-induced ALI mouse model does not fully recapitulate the immunopathology of SARS-CoV-2 infection, we deliberately chose this non-viral model to isolate zapnometinib’s direct immunomodulatory effects from its antiviral activity. In a viral infection model, reduced cytokine and chemokine expression could arise indirectly from decreased viral load, making it challenging to distinguish primary immunomodulatory effects from secondary consequences of antiviral action. The ALI model, with its pathogen-independent cytokine induction, allowed us to demonstrate zapnometinib’s direct anti-inflammatory potential without this confounding factor. Nevertheless, we acknowledge that this approach has limitations in faithfully modelling the complexity of viral immunopathology. Future studies using SARS-CoV-2 infection models will be important to confirm and extend these findings in a more clinically relevant setting. In addition to pathogen infection, ALI is an inflammatory condition that can be triggered by infections, sepsis, trauma, ischemia, or reperfusion. The most severe manifestation is ARDS (acquired respiratory distress syndrome), a hallmark of severe COVID-19 (75). In the ALI model, excessive inflammatory response was induced by bacterial LPS stimulation. Zapnometinib treatment led to a massive reduction in hyperinflammatory cytokine responses, highlighting its immunomodulatory potency *in vivo*. In addition, we assessed zapnometinib’s effectiveness in a primary human cell model, namely freshly isolated PBMCs, where a similar cytokine reduction was observed, confirming its immunomodulatory effect. A limitation of the ex vivo PBMC experiments is the small number of donors (n=2), which may limit generalizability. However, the highly consistent cytokine and chemokine response profiles across both donors support the mechanistic validity of the observed immunomodulatory effects of zapnometinib, in line with previously published MEK inhibitor data.

These pre-clinical findings were confirmed in the RESPIRE study, showing significant reductions in MCP-1, MIP-1a and IFN-γ levels. However, the implications for IFN-γ remain uncertain as it has dual roles as both an inducer and a regulator of inflammation (76). Notably, IL-8/CXCL8 levels showed a slight though not statistically significant increase with zapnometinib treatment. CXCL8, together with its receptors CXCR1/2, triggers neutrophil activity to combat inflammation during bacterial infections (77, 78). Although the overserved increase did not reach statistical significance, this trend may suggest a potentially beneficial role for CXCL8 in modulating immune responses during viral-induced inflammation. Overall, these findings highlight zapnometinib’s significant anti-inflammatory potential.

In recent years, many studies have highlighted the great potential of MEK inhibitors as immunomodulators (23, 79, 80). These inhibitors not only reduce excessive cytokine responses but also modulate the adaptive immune response for improved antiviral efficacy. Severe and moderate stages of COVID-19 are associated with lymphocyte depletion (81). In the RESPIRE study, we demonstrated that treatment with zapnometinib resulted in an increase in the number of lymphocytes, including T cells and antibody-producing plasma B cells, while the quantity of memory B cells was reduced. This shift may suggest an enhanced availability of plasma cells at the expense of memory B cells, potentially supporting a more active antibody response in treated patients (82). Given the scope of this manuscript as an integrated summary of preclinical models and first clinical trial results, we focus here on these initial findings. A more detailed analysis of T cell subpopulations from the RESPIRE trial will be presented in a dedicated follow-up publication. In summary, the positive effect on the adaptive immune response also shows the great potential of treating respiratory viral infections by a MEK inhibitor.

In some patients in the RESPIRE study, glucocorticoids were administered as part of the standard of care. Dexamethasone, a glucocorticoid commonly used to treat severe COVID-19, suppresses cytokine production by mainly targeting NF-κB signaling, as shown in animal models of LPS-induced ALI and in human PBMCs (83, 84). Additionally, dexamethasone down-regulates type I interferons (IFNs), which are crucial in the initial defense against severe viral respiratory infections (75, 85, 86). A drawback of glucocorticoids is their immunosuppressive nature, which can compromise host immunity and prolong viral clearance. In addition, tocilizumab and baricitinib, two immunomodulatory agents recommended for severe COVID-19 treatment, have shown effectiveness in attenuating multiple cytokines and chemokines in animal models of ALI and in human PBMCs (87). The study did not conclusively show a direct impact on interference with type I interferon (IFN). However, as baricitinib inhibits NF-κB, it is presumed that it may influence type I IFN expression since NF-κB plays a crucial role in their regulation (88, 89). In contrast to the NF-κB cascade, inhibiting the Raf/MEK/ERK signaling pathway, as demonstrated in this and previous studies, does not attenuate type I IFNs and downstream IFN-regulated genes (36). This suggests that zapnometinib treatment has a notable advantage over dexamethasone, tocilizumab, and baricitinib, as it does not diminish the type I IFN response. The potential to modulate an excessive proinflammatory cytokine and chemokine response remains comparable.

Overall, the study underscores the noteworthy therapeutic potential of zapnometinib as a novel and broadly effective approach. In recent years, there have been emerging strategies employing HTAs as broad-spectrum antiviral agents, with particular emphasis on repurposing kinase inhibitors. However, most substances have only been tested in proof-of-concept studies so far (90, 91). Our dual effect represents a distinct advantage over current therapies, which typically target either the virus itself or the hyperinflammatory response. In contrast, by inhibiting the Raf/MEK/ERK signaling pathway, zapnometinib exhibits both a reduction in viral load and a modulation rather than complete suppression of the immune response (39, 92). This sets the drug apart from most, if not all, COVID-19 treatments currently licensed or in development.

The findings presented here align with our earlier studies (36) which emphasize the potential value of zapnometinib as a standalone treatment or in combination with other drugs targeting various stages of viral infection and disease progression. Such combinations may represent a promising strategy for COVID-19 control. Furthermore, zapnometinib’s implications extend beyond COVID-19, as evidenced by its recent orphan drug designation by the FDA for hantavirus infections and the initiation of a phase 2 clinical trial for influenza. The robust correlation between preclinical and clinical data for SARS-CoV-2 and COVID-19 suggests a potential treatment benefit for influenza (28, 29, 93). Collectively, these findings underscore the broad-spectrum anti-infective potential of zapnometinib against acute viral infections. Importantly, the clinical impact of zapnometinib has been reported in detail in our companion publication (42), where treatment was associated with improved clinical scores and accelerated symptom resolution in hospitalized COVID-19 patients, in addition to the virological and immunological effects described here.

## Data Availability

The data that support the findings of this study are available upon reasonable request from the corresponding author.
