# Supplementary material for: Antiviral and immunomodulatory effect of zapnometinib in animal models and hospitalized COVID-19 patients
Source: Front Immunol. 2025 Sep 22;16:1631721. doi: 10.3389/fimmu.2025.1631721 (PMC12498288; doi:10.3389/fimmu.2025.1631721)
Supplement: Supplementary file 1 [file DataSheet1.docx]

Supplementary Material

**Antiviral and immunomodulatory effect of zapnometinib in animal models and hospitalized COVID-19 patients**

This file includes:

**Supplementary Materials and Methods**  page 2 - 4

**Supplementary Figures 1 – 4** page 5 - 9

**Supplementary Table 1 – 3** page 10 - 16

**Supplementary References**  page 16

**Supplementary Materials and Methods**

**Cytotoxicity assessment and CC_50_ determination**

In parallel to the *in vitro* antiviral assays, cytotoxicity was analyzed for the corresponding assay conditions using LDH release assay (CyQUANT^TM^ LDH cytotoxicity assay Kit, # 16270972, Fisher Scientific) according to manufacturer´s protocol. Briefly, 3 µL of cell culture medium supernatant after compound treatment on Vero cells or Vero E6 cells (as described in section “Virospot reduction assay”) were diluted in 297 µL LDH storage buffer. The samples were transferred to a 96-well plate, mixed and incubated with the reaction mix for 30 minutes and analyzed by colorimetric read-out in a microplate reader at 490 nm. Maximum LDH release control were cells treated with lysis buffer as described in the manufacturer´s protocol.

The concentrations used in the assays (up to 100 µM) did not reach 50% cytotoxicity, so CC_50_ values were further determined in another experiment using the CyQUANT™ LDH Cytotoxicity Kit (Cat# C20301, Fosher Scientific) following the manufacturer’s instructions. In short, 2x10^4^ Calu-3 cells were seeded per well in 96-well cell culture plates in IMDM medium containing 10% FBS, 100 units/mL penicillin, and 100 µg/mL streptomycin two days before treatment. Prior to the treatment, the medium was removed, and the cells were washed once with PBS. The cells were subsequently treated in quadruplicates with 600, 500, 400, 350, 300, 250, 200, 150, or 100 µM zapnometinib dissolved in DMSO and diluted in IMDM medium containing 5% FBS, 100 units/mL penicillin, and 100 µg/mL streptomycin. The DMSO concentration upon treatment was 1%. A DMSO control was supplied with 1% DMSO (without zapnometinib). An untreated control (spontaneous LDH release control) was supplied with cell culture medium (5% FBS). Viability assessment with LDH assay was performed after 24 and 48 h treatment. Therefore, 10 µL 10X Lysis Buffer were added to one well of each quadruplicate treatment set (maximum LDH release control) and the plates were incubated 45 min at 37 °C and 5% CO_2_. 50 µL Reaction Mix were added to the wells of a fresh 96-well plate, and 50 µL cell culture supernatant was transferred from the treated cells to the wells containing Reaction Mix. After 30 min incubation in the dark at RT, 50 µL stop solution were added to the wells and the absorbance was read at 490 nm and 680 nm. The absorbance measured at 680 nm was subtracted from the absorbance at 490 nm and the %Viability was calculated using the following formula:

%Viability = 100-((compound-treated LDH release - spontaneous LDH release)/(Maximum LDH release - spontaneous LDH release))*100.

The CC_50_ was determined by plotting the viability values (on the y axis) against the corresponding log_10_ transformed zapnometinib concentrations (on the x axis) and interpolating the zapnometinib concentration at 50 % viability using the “interpolate a standard curve (Sigmoidal, 4PL, X is log(concentration)” function of the GraphPad Prism software v9.

**Cytokine and chemokine analysis**

Eleven- to thirteen-week-old male Syrian hamsters (Janvier, France) with a body weight of 113-148 g were infected on day 0 intranasally with SARS-CoV-2 (1x10^3^ median tissue culture infectious dose (TCID_50_) BetaCoV/Munich/BavPat1/2020) in a total volume of 0.1 mL PBS divided between the nostrils. Zapnometinib (100 mg/kg) was administered p.o. at 4 or 24 h p.i. and the animals in both groups were treated thereafter with 75 mg/kg zapnometinib once daily. The animals in the control group received vehicle (5% DMSO, 15% Kolliphor, 80% PBS) 4 h p.i. and thereafter once daily. Serum samples were collected from hamsters before infection (0 dpi) and at 4 dpi, and the levels of different cytokines and chemokines (IL1-β, TNF-α, IL-6, and IP-10) were analyzed by ELISA (Assay Genie, Ireland). ELISA was performed according to the manufacturer’s instructions. Briefly, the serum samples and standards were added to ELISA plates precoated with specific antibodies against the cytokines of interest. Biotinylated detection antibodies binding to the cytokines of interest were added to the wells followed by a horseradish peroxidase (HRP)-streptavidin conjugate binding to the biotinylated detection antibodies. Unbound cytokines, detection antibodies, and HRP-streptavidin conjugate were removed by washing. Tetramethylbenzidine (TMB) substrate was added and the color change in the wells was used as readout for the ELISA. The optical density (OD) at 450 nm was read. Cytokine and chemokine concentrations were calculated following the manufacturer’s instructions for interpolating unknown concentrations from a standard curve.

RNA samples derived from hamster lung tissue at 4 dpi from the efficacy study (animals were infected and treated as described above) provided by Viroclinics Xplore were subjected to gDNA digestion using RNase-free DNase I set (Qiagen, Hilden, Germany) and cleaned with RNeasy Mini kit (Qiagen) according to the manufacturer’s instruction. Five primer sets for the TNF-α, IL-6, IL-1β, IP-10 and RPL-18 genes were designed according to the sequences available in GenBank. After evaluating the specificity of the primers by melting curve analysis, in one-step RT-qPCR SYBR Green assay, and agarose gel electrophoresis, real-time one-step multiplex RT-qPCR was carried out using an ultrafast QuantiNova multiplex RT-qPCR kit according to the manufacturer’s instructions to evaluate alterations in gene expression. The data were analyzed according to the ΔΔCq method, and relative expression was calculated based on the formula “relative expression = 2^Cq(ref) - Cq(target)^”.

Statistical analyses were performed using GraphPad Prism software (version 8) (GraphPad Software, San Diego, California, USA). Differences in cytokine and chemokine expression were analyzed using ANOVA, details are described in the corresponding figure legends. P values < 0.05 were considered statistically significant.

**Candidate biomarker analysis**

The candidate protein biomarkers were quantified via specific peptides analyzed by immunoaffinity liquid chromatography tandem mass spectrometry (IA-LC-MS/MS) assays. For proteolysis, 10 μL of each serum sample was mixed with 50 μL of digestion buffer (100 mM triethanolamine and 0.5% (w/v) n-octylglucoside (NOG)), and proteolysis reagents were added to achieve a final sample volume of 100 µL. The proteolysis procedure included denaturation at 99 °C for 5 minutes, reduction in 5 mM tris(2-carboxyethyl)-phosphine at 22 °C for 5 minutes, alkylation in 10 mM iodoacetamide at 22 °C for 20 minutes and an overnight enzymatic fragmentation at 37 °C with a trypsin-to-protein ratio of 1:20 (assuming a concentration of 60 mg/mL protein in serum). The proteolysis was inactivated by heating at 99 °C for 5 minutes and addition of phenylmethanesulfonylfluoride to a final concentration of 10 mM. For subsequent peptide-targeted immunoaffinity enrichment, a mixture of stable isotope-labeled standard (SIS) peptides with known concentrations was added to the proteolyzed samples as internal standards (Intavis AG, Tuebingen, Germany). Peptide-specific antibodies were generated as described previously (1) and added to the proteolyzed samples for immunoaffinity enrichment. Semiautomated immunoprecipitation was performed with a magnetic particle processor (KingFisher 96, Thermo Fisher Scientific, Waltham, USA) as described previously (2). Chromatography and mass spectrometry were performed as previously described (3).

The following peptide transitions were monitored in the mass spectrometer : HGGTIPVVPTAEFQDR (GLDH), endogenous 575.30 (p+++) ® 482.23 (y8+) and SIS 578.63 (p+++) ® 487.23 (y8+), collision energy (CE) 19.6 V; GEHPGLSIGDVAK (HMGB1), endogenous 640.34 (p++) ® 956.54 (y10+), SIS 644.34 (p++) ® 964.56 (y10+), CE 33.4 V; and HFTDLVAIQNK (SELP), endogenous 643.35 (p++) ®573.34 (y5+), SIS 647.36 (p++) ® 581.45 (y5+), CE 36.5 V.

Subsequently, the acquired MRM data files were imported into Skyline software version 19.1 (4). The peak areas of endogenous and SIS peptides were extracted. The unknown analyte concentration was calculated via referencing the peak area of endogenous signal to the spiked internal standard with a known concentration. Protein concentrations were calculated by converting the quantified peptide concentration (fmol/µL) into a mass concentration (ng/mL) considering the molecular weight of the corresponding protein. The molecular weight of each protein was calculated according to the amino acid sequence, which was inferred from UniProt release 2019_04 (FASTA sequence), and the theoretical molecular weight was calculated using the corresponding amino acid sequence with the online tool ProtParam (5).

**Supplementary Figure 1**


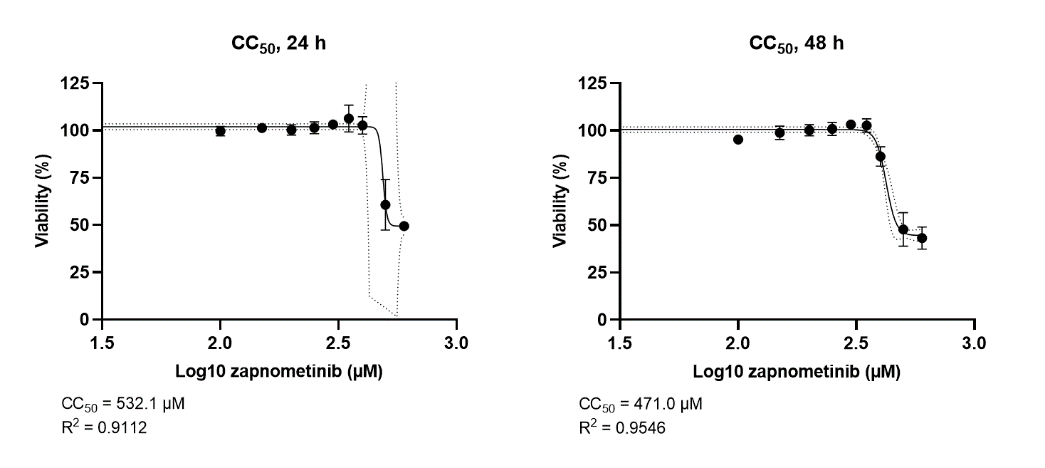


**Supplementary Figure 1. *In vitro* CC_50_ assessment of zapnometinib.**

The 50% cytotoxic concentration (CC_50_) was determined on Calu-3 cells in a LDH cytotoxicity assay. The cells were treated with 100-600 µM zapnometinib or were left untreated (control). Viability assessment with LDH assay was performed after 24 and 48 h treatment. The %Viability was calculated and the CC_50_ was determined by plotting the viability vs. the zapnometinib concentration. The data is shown as mean with standard deviation. n_(24 h, 200-500 µM and control)_ = 9; n_(24 h, 100, 150, and 600 µM)_ = 6; n_(48 h, 200-600 µM and control)_ = 9; n_(48 h, 100 and 150 µM)_ = 6.

**Supplementary Figure 2**

**
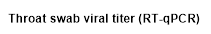
**

**A**

**
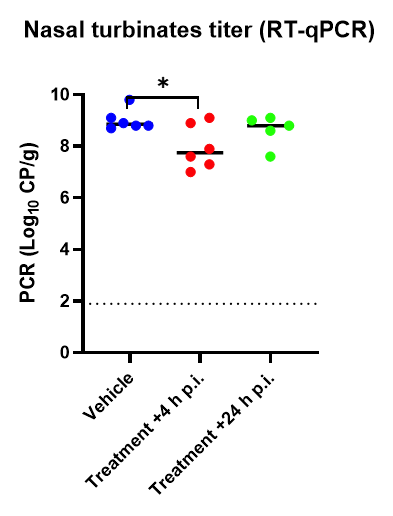
B**

**Supplementary Figure 2. Viral titers analyzed by RT-qPCR in samples of Syrian hamsters infected with SARS-Cov-2 and treated with zapnometinib**

**A.** Infectious viral titers in throat swabs collected at 2, 3 and 4 dpi of control (vehicle-treated) and zapnometinib-treated SARS-CoV-2-infected hamsters expressed as Log_10_ genome copies per g determined by RT-qPCR **B**. Infectious viral loads in nasal turbinates collected from control (vehicle-treated) and zapnometinib-treated SARS-CoV-2-infected hamsters at 4 dpi expressed as Log_10_ copies per g determined by RT-qPCR. The dashed lines represent the lower limit of detection (1.9 log_10_ CP/mL). Mean and individual values are shown. The data were analyzed by one-way ANOVA. * *p < 0.05*.

**Supplementary Figure 3**

**A**


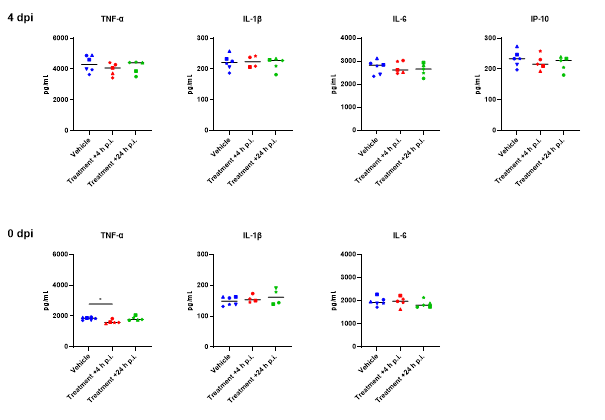


***
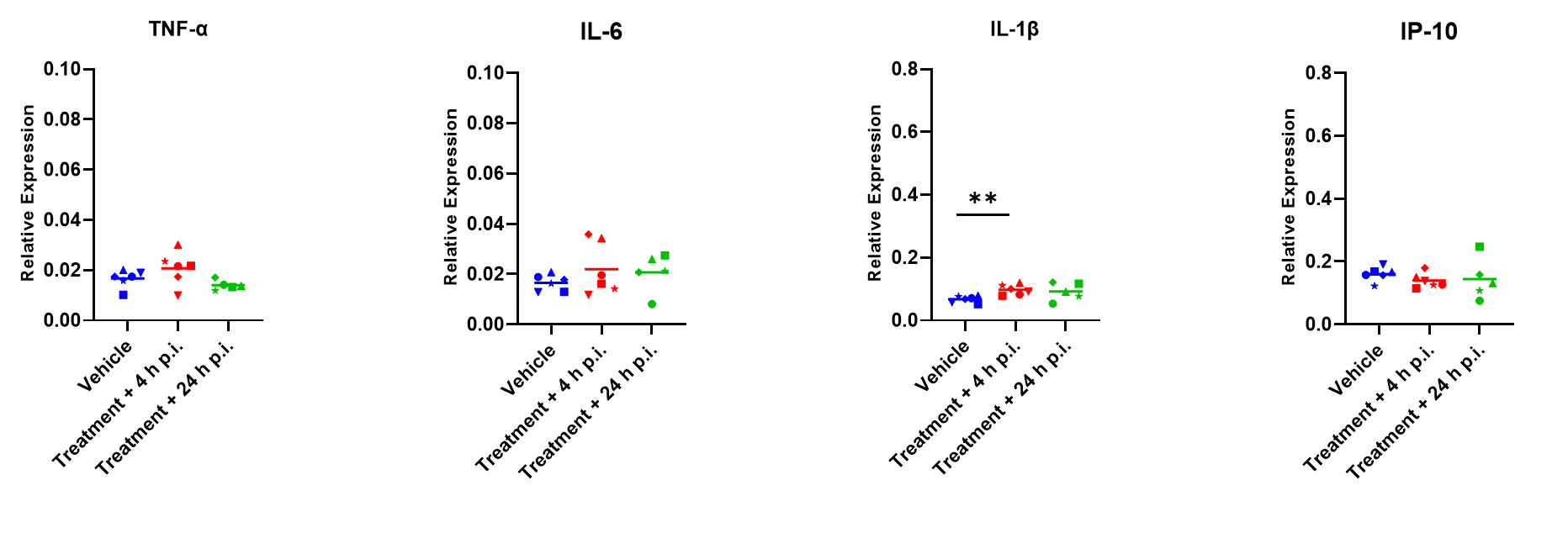
*B**

**Supplementary Figure 3. Cytokine and chemokine levels in *S*yrian hamsters before and after SARS-CoV-2 infection and zapnometinib treatment.**Animals were infected on day 0 (0 dpi) with SARS-CoV-2. Zapnometinib (100 mg/kg) was administered p.o. at 4 or 24 h p.i. and the animals in both groups were treated thereafter with 75 mg/kg zapnometinib once daily, control group received vehicle (5% DMSO, 15% Kolliphor, 80% PBS) 4 h p.i. and thereafter once daily. **A**. Cytokine levels in serum samples collected at 0 dpi (before infection and treatment, bottom row) and 4 dpi (top row) from vehicle-treated and zapnometinib-treated Syrian hamsters from the efficacy study analyzed by ELISA are shown. The treatment conditions described below the graphs refer to the treatment that the animals received after infection. The cytokine level of each animal and the mean values of each treatment group are presented (horizontal line). n_(vehicle)_ = 6; n_(Treatment +4 h p.i.)_ = 5 or 4 (n = 4 for analysis of IL-1β levels at 4 dpi and 0 dpi); n_(Treatment +24 h p.i.)_ = 5 or 4 (n = 4 for analysis of IL-1β levels at 0 dpi). Differences between the vehicle and treatment groups were not statistically significant at 4 dpi (Brown-Forsythe ANOVA with the Dunnett T3 correction). Only the TNF-α level at 0 dpi was significantly different between the group that would later be treated with vehicle and the group that would later be treated with zapnometinib at 4 h p.i. (adjusted *p value* = *0.0415*; Brown-Forsythe ANOVA with the Dunnett T3 correction).

**B.** Relative mRNA expression of different cytokines and chemokines in lung tissue of Syrian hamsters after SARS-CoV-2 infection and zapnometinib treatment**.** mRNA levels were determined using one-step multiplex RT-qPCR in lung samples collected after sacrifice at 4 dpi. The data were analyzed based on the ΔΔCt method, and gene expression was normalized to the expression of the RPL-18 gene as a housekeeping gene. The data are presented as individual values with the mean values of the 6 (Vehicle and Treatment +4 h p.i.) or 5 animals (Treatment +24 h p.i.) per group (n = 3 technical replicates). P values of replicate 2^(-ΔCt) expression values for each gene between the control and treatment groups were calculated by one-way ANOVA and the Kruskal-Wallis test. ** *p < 0.01*

**Supplementary Figure 4**

**
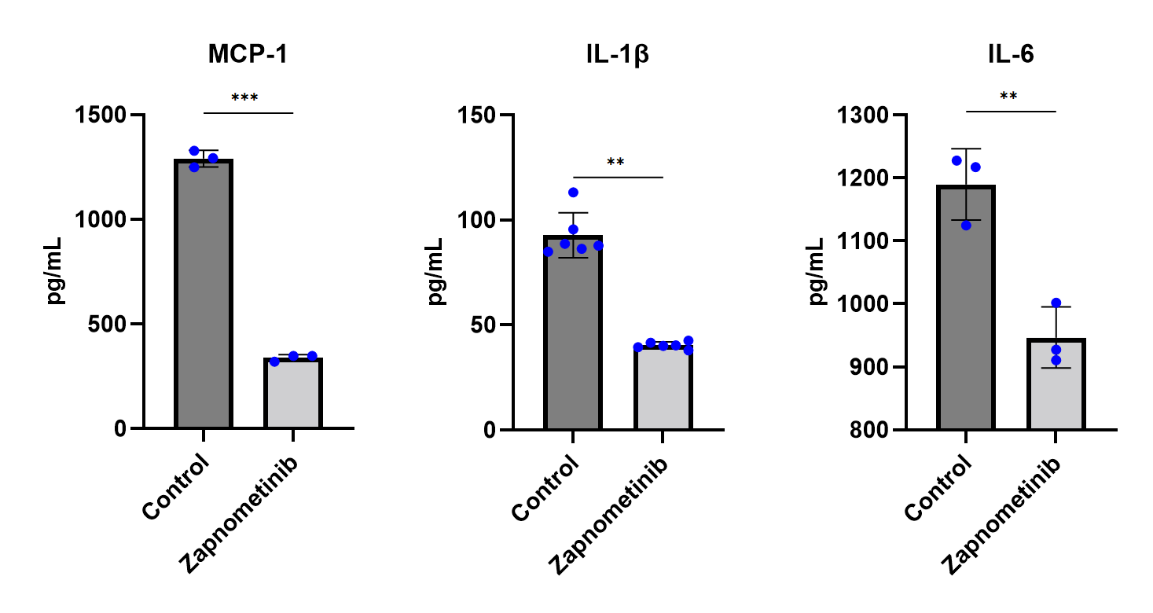
**

**Supplementary Figure 4.** **The levels of cytokines and chemokines associated with COVID-19 are reduced after treatment with zapnometinib in human PBMCs.**

Reduction in cytokine expression after zapnometinib treatment in LPS-stimulated human PBMCs from a second healthy donor. Cells were simultaneously stimulated with 1 µg/mL LPS from *E. coli* O55:B5 and treated with 10 µg/mL zapnometinib for 6 h. The expression of cytokines in the supernatants was analyzed by ELISA. The data are presented as individual values (dots) and the mean ± SD of technical replicates. n_(MCP-1, IL-6)_ = 3; n_(IL-1β)_ = 6. The differences in the levels of all cytokines of interest between the control and zapnometinib-treated groups were analyzed with unpaired t-test with Welch’s correction if data were normally distributed, otherwise with Mann-Whitney test. ** *p > 0.01;* *** *p > 0.001.*

**Supplementary Table 1**: **Oligonucleotide sequences of primers and probes used for the RT–qPCR assays**

| Target gene | Primer/Probe | Sequence (5`-3`) | Reference |
| --- | --- | --- | --- |
| TNF | ha_TNF-F | CCCACGTTGTAGCAAACCAC | This study  (Hamster) |
|  | ha_TNF-R | TTTGAGAGACATGCCGTTGG |  |
|  | ha_TNF-P | [6FAM] AGCCATCGTGCCAATGCCCT [BHQ1] |  |
| IL-6 | ha_IL-6-F | TCAGAGCACCATCAAAACCC |  |
|  | ha_IL-6-R | TTGGCCACTCCTTTTGTGAC |  |
|  | ha_IL-6-P | [6FAM] AGCCCAACCTCCAAGGCCAT [BHQ1] |  |
| IL-1b | ha_IL-1b-F | TCATCTTTGAAGAAGAGCCCATC |  |
|  | ha_IL-1b-R | TGTTCTGTCCGTTGAGATGGAG |  |
|  | ha_IL-1b-P | [6FAM] TCGTGCTGTCTGACCCCTGTGA [BHQ1] |  |
| IP-10 | ha_IP-10-F | ATGGTCACATCAGCTGCTATCC |  |
|  | ha_IP-10-R | AGTTGGGGACTCTTGTCACTG |  |
|  | ha_IP-10-P | [6FAM] TTCTCCAGGACGATGGGCAGCT [BHQ1] |  |
| RPL-18 | ha_RPL-18-F | CAAGATCCTCACCTTTGACCAG |  |
|  | ha_RPL-18-R | GAGCGGACATAGGGTTTGGTA |  |
|  | ha_RPL-18-P | [CY5] TGGCACTGTGCTCCTGTCTGGT [BHQ2] |  |
| E gene | E_Sarbeco_F  E_Sarbeco_R  E_Sarbeco_P1 | ACAGGTACGTTAATAGTTAATAGCGT  ATATTGCAGCAGTACGCACACA  ACACTAGCCATCCTTACTGCGCTTCG | Corman et al. 2020 |
| SARS-CoV-2 2019 & Omicron variant | | | |
| N gene | 2019-CoV2-N1F | AACACAAGCTTTCGGCAGAC | This study |
|  | 2019-CoV2-N1R | ATTCCGAAGAACGCTGAAGC |  |
|  | 2019-CoV2-N1P | [6FAM] ACATTGGCCGCAAATTGCACAA [BHQ1] |  |
| HCoV-229E | | | |
| N gene | HCoV229E-N1F | AAAGCCACGGTGGAAAAGAC | This study |
|  | HCoV229E-N1R | AACACCATTGGCCACAACAC |  |
|  | HCoV229E-N1P | [6FAM] TGGCCCCAGAGACCTTGACCA [BHQ1] |  |
| HCoV-OC43 | | | |
| NS2 gene | HCoVOC43-NS2F | ATTGGCCATTGCACCATAGC | This study |
|  | HCoVOC43-NS2R | TTCAAGTCTAGCCGGTGATGAG |  |
|  | HCoVOC43-NS2P | [6FAM] TCACGGATGCAGCACTGTCCA [BHQ1] |  |

**Supplementary Table 2**: **Cytotoxicity run (LDH release assay) in parallel to *in vitro* antiviral assay (Virospot reduction assay)**

| **Cells\Conc.(µM)** | **100.00** | **75.00** | **50.00** | **25.00** | **12.50** | **6.25** | **3.13** | **1.56** | **0.78** | **0.39** |
| --- | --- | --- | --- | --- | --- | --- | --- | --- | --- | --- |
| Vero 24 h 5% FBS | 1.44 | 0.83 | 0.49 | 0.12 | 0.31 | 0.02 | -0.37 | 0.41 | 0.41 | 0.35 |
| Vero E6 24 h 5% FBS | 0.26 | 0.24 | 0.02 | -0.04 | 0.09 | 0.13 | 0.39 | 0.15 | 0.17 | 0.22 |

Data is presented as %LDH release compared to maximum LDH release (cells treated with lysis buffer) set as 100%, each compound concentration was tested in duplicate per cell line

**Supplementary Table 3**: **Mean viral titer and mean change from baseline (Day 0) over time (Day 3 – Day 30) in SARS-CoV-2 RNA titer from nasopharyngeal samples (all randomized MITT population) from patients of the RESPIRE clinical trial treated either with zapnometinib or placebo D1 - D6**

| **Nasopharyngeal** | | |  |  |  |  |  |  |  |  |  |
| --- | --- | --- | --- | --- | --- | --- | --- | --- | --- | --- | --- |
| **Visit** | **Zapnometinib** | | |  | **Placebo** | | |  | **Zapnometinib - Placebo** | | |
|  | n | Baseline Mean | Mean Change^a^ (SD) | | n | Baseline Mean | Mean Change^a^ (SD) | | Diff. | (95% CI) | p-value |
| **SARS-CoV-2 RNA titer (log10 copies/ml) in participants overall** | | | | | | | | | | | |
| Baseline | 31 | 5.55 |  |  | 30 | 5.02 |  |  |  |  |  |
| Day 3 | 31 | 4.78 | 0.77 | (1.51) | 30 | 3.88 | 1.14 | (1.37) | -0.36 | -1.104 to 0.375 | 0.1639 |
| Day 5 | 27 | 3.78 | 1.79 | (1.64) | 29 | 3.27 | 1.68 | (1.48) | 0.11 | -0.728 to 0.950 | 0.3960 |
| Day 8 | 24 | 2.78 | 2.84 | (1.59) | 28 | 2.70 | 2.32 | (1.59) | 0.53 | -0.349 to 1.404 | 0.1162 |
| Day 11 | 22 | 2.42 | 3.16 | (1.82) | 22 | 2.18 | 2.87 | (1.39) | 0.29 | -0.700 to 1.273 | 0.2803 |
| Day 15 | 25 | 2.16 | 3.55 | (1.79) | 28 | 1.89 | 3.06 | (1.57) | 0.49 | -0.443 to 1.428 | 0.1477 |
| Day 30 | 24 | 1.93 | 3.89 | (1.60) | 23 | 1.76 | 3.14 | (1.61) | 0.75 | -0.195 to 1.687 | 0.0586 |
| **SARS-CoV-2 RNA titer non omicron (log10 copies/ml) in participants overall** | | | | | | | | | | | |
| Baseline | 17 | 4.72 |  |  | 17 | 4.62 |  |  |  |  |  |
| Day 3 | 17 | 3.67 | 1.05 | (1.42) | 17 | 3.43 | 1.19 | (1.46) | -0.14 | -1.148 to 0.863 | 0.3874 |
| Day 5 | 15 | 2.85 | 1.72 | (1.58) | 16 | 2.85 | 1.62 | (1.48) | 0.10 | -1.025 to 1.226 | 0.4280 |
| Day 8 | 13 | 2.21 | 2.50 | (1.52) | 15 | 2.14 | 2.43 | (1.38) | 0.07 | -1.065 to 1.210 | 0.4482 |
| Day 11 | 11 | 2.43 | 2.03 | (1.64) | 12 | 2.16 | 2.74 | (1.17) | -0.71 | -1.968 to 0.551 | 0.1263 |
| Day 15 | 13 | 2.24 | 2.45 | (1.65) | 15 | 2.08 | 2.35 | (1.14) | 0.10 | -1.031 to 1.234 | 0.4269 |
| Day 30 | 12 | 2.00 | 2.83 | (1.27) | 12 | 1.90 | 2.55 | (1.12) | 0.29 | -0.736 to 1.292 | 0.2874 |
| **SARS-CoV-2 RNA titer omicron (log10 copies/ml) in participants overall** | | | | | | | | | | | |
| Baseline | 14 | 7.10 |  |  | 13 | 5.54 |  |  |  |  |  |
| Day 3 | 14 | 6.65 | 0.44 | (1.60) | 13 | 4.47 | 1.07 | (1.31) | -0.63 | -1.786 to 0.528 | 0.1366 |
| Day 5 | 12 | 5.51 | 1.87 | (1.78) | 13 | 3.79 | 1.75 | (1.54) | 0.12 | -1.261 to 1.510 | 0.4269 |
| Day 8 | 11 | 4.09 | 3.25 | (1.64) | 13 | 3.35 | 2.19 | (1.77) | 1.06 | -0.382 to 2.505 | 0.0707 |
| Day 11 | 11 | 3.07 | 4.29 | (1.18) | 10 | 2.19 | 3.04 | (1.68) | 1.25 | -0.100 to 2.608 | **0.0336** |
| Day 15 | 12 | 2.86 | 4.74 | (1.02) | 13 | 1.66 | 3.88 | (1.64) | 0.87 | -0.264 to 1.995 | 0.0628 |
| Day 30 | 12 | 2.60 | 4.94 | (1.13) | 11 | 1.61 | 3.78 | (1.85) | 1.16 | -0.209 to 2.530 | **0.0457** |

Analysis only includes patients with baseline nasopharyngeal SARS-CoV-2 RNA titer ≥ 500 copies/ml. Baseline results below this limit were excluded from the analysis.

^a^Mean change from baseline values are based on the measurements from patients with values at both baseline and at the time point assessed.

**Supplementary Table 4**: **List of genes from the RT² Profiler™ Mouse Cytokines & Chemokines array (PAMM-150ZR, Qiagen) analyzed in the ALI mouse model and presented in the volcano plot (Fig. 5a)**

See separate file.

**Supplementary Table 5**: **Standard of care (SOC) treatment of patients from the RESPIRE clinical trial used for the analysis of virological or immunological parameters**

| **SOC** | **SOC-Type** | **Treatment** |
| --- | --- | --- |
| None | Other | Zapnometinb |
| None | Other | Zapnometinb |
| None | Other | Zapnometinb |
| CASIRIVIMAB+IMDEVIMAB | Other | Zapnometinb |
| CASIRIVIMAB+IMDEVIMAB | Other | Zapnometinb |
| CASIRIVIMAB+IMDEVIMAB | Other | Zapnometinb |
| None | Other | Zapnometinb |
| DEXA | Steriode | Zapnometinb |
| DEXA, TLZ | Steriode | Zapnometinb |
| RDV, MPRED | Steriode | Zapnometinb |
| MPRED | Steriode | Zapnometinb |
| BUDE | Steriode | Zapnometinb |
|  |  |  |
| CASIRIVIMAB+IMDEVIMAB | Other | Placebo |
| CASIRIVIMAB+IMDEVIMAB | Other | Placebo |
| CASIRIVIMAB+IMDEVIMAB | Other | Placebo |
| CASIRIVIMAB+IMDEVIMAB | Other | Placebo |
| CASIRIVIMAB+IMDEVIMAB | Other | Placebo |
| CASIRIVIMAB+IMDEVIMAB | Other | Placebo |
| RDV, DEXA, MPRED | Steriode | Placebo |
| PRED | Steriode | Placebo |
| DEXA, BUDE | Steriode | Placebo |
| PRED | Steriode | Placebo |
| DEXA, TLZ | Steriode | Placebo |
| DEXA | Steriode | Placebo |

DEXA = Dexamethasone; TLZ = Tocilizumab; RDV = Remdesivir; MPRED = Methylprednisolone; BUDE = Budesonid; PRED = Prednisolone

**Supplementary References**

1 Hoeppe, S. *et al.* Targeting peptide termini, a novel immunoaffinity approach to reduce complexity in mass spectrometric protein identification. *Mol Cell Proteomics* **10**, M110 002857, doi:10.1074/mcp.M110.002857 (2011).

2 Steinhilber, A. E. *et al.* Mass Spectrometry-Based Immunoassay for the Quantification of Banned Ruminant Processed Animal Proteins in Vegetal Feeds. *Anal Chem* **90**, 4135-4143, doi:10.1021/acs.analchem.8b00120 (2018).

3 Belghit, I. *et al.* Future feed control – Tracing banned bovine material in insect meal. *Food Control* **128**, 108183, doi:<https://doi.org/10.1016/j.foodcont.2021.108183> (2021).

4 Pino, L. K. *et al.* The Skyline ecosystem: Informatics for quantitative mass spectrometry proteomics. *Mass Spectrom Rev* **39**, 229-244, doi:10.1002/mas.21540 (2020).

5 UniProt, C. UniProt: the universal protein knowledgebase in 2021. *Nucleic Acids Res* **49**, D480-D489, doi:10.1093/nar/gkaa1100 (2021).
